# Simultaneous Determination of Aristoloxazines, Aristolochic Acids, and Aristolactams Using HPLC–Fluorescence Detection with a Post-column Microreactor: Application in Identifying New Aristoloxazines

**DOI:** 10.1021/acs.jafc.5c11092

**Published:** 2025-10-22

**Authors:** Man-Lung Chin, Martin M.F. Choi, Rongbiao Tong, Nikola M. Pavlović, Wan Chan

**Affiliations:** † Department of Chemistry, 58207 The Hong Kong University of Science and Technology, Clear Water Bay, Kowloon 999077, Hong Kong; ‡ Medical Faculty, University of Niš, Nis 18000, Serbia; § InnovationCenter, Universityof Niš, Univerzitetskitrg 2, Niš 18106, Serbia; ∥ KidneyaTherapeutics, Belgrade 11070, Serbia

**Keywords:** aristoloxazines, aristolochic acid, aristolactams, HPLC−FLD, post-column microreactor

## Abstract

Aristoloxazines (AXs), aristolochic acids (AAs), and aristolactams (ALs) are potent genotoxins found in *Asarum* and *Aristolochia* plants, many of which are commonly used as herbal medicines. Emerging evidence indicates that these compounds contaminate arable soil during the cultivation of *Aristolochiaceae* herbs. Currently, no method exists for their simultaneous detection. In this study, we developed a high-performance liquid chromatography (HPLC)-based method for their determination. This method employs an iron powder-packed microreactor to convert non-fluorescent AXs and AAs into naturally fluorescent ALs, enabling fluorescence detection after HPLC separation. After being validated against LC–MS/MS analysis, the method was applied to quantify these genotoxins in herbal and soil samples, detecting AXs at concentrations as high as hundreds of μg/g in some *Asarum* samples. Given that *Asarum* plants are widely used in herbal medicine, these results reveal previously unrecognized human exposure to genotoxins that warrants the attention of both the general public and regulatory agencies.

## Graphical abstract

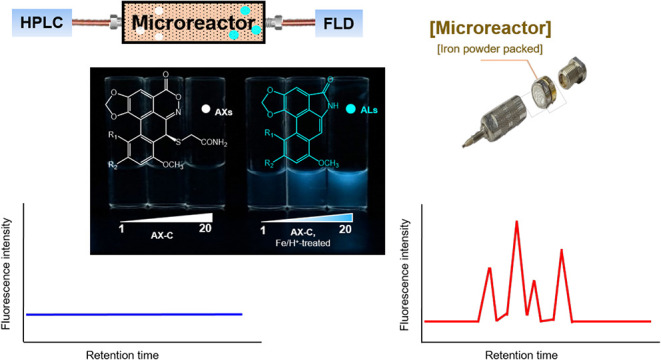

## Introduction

Aristoloxazines (AXs; Figure 1) are a recently identified family of sulfur-containing phytochemicals found in certain *Asarum* and *Aristolochia* plants.^1–4^ These plants have a long history of use in herbal medicine for treating various ailments, including skin diseases,^5–7^ coughs,^6,8–10^ and pneumonia.^11^ Notably, *Asari Radix et Rhizoma* (*Xixin*), while known to contain low levels of the carcinogenic and nephrotoxic aristolochic acids (AAs; Figure 1),^5,6,10,12^ remains readily available in herbal stores across Hong Kong, Taiwan, and mainland China. Furthermore, *Xixin* is a key ingredient in a Chinese herbal prescription endorsed by the National Administration of Traditional Chinese Medicine for treating severe acute respiratory syndrome coronavirus 2 (SARS-CoV-2).^13^

**Figure 1:**
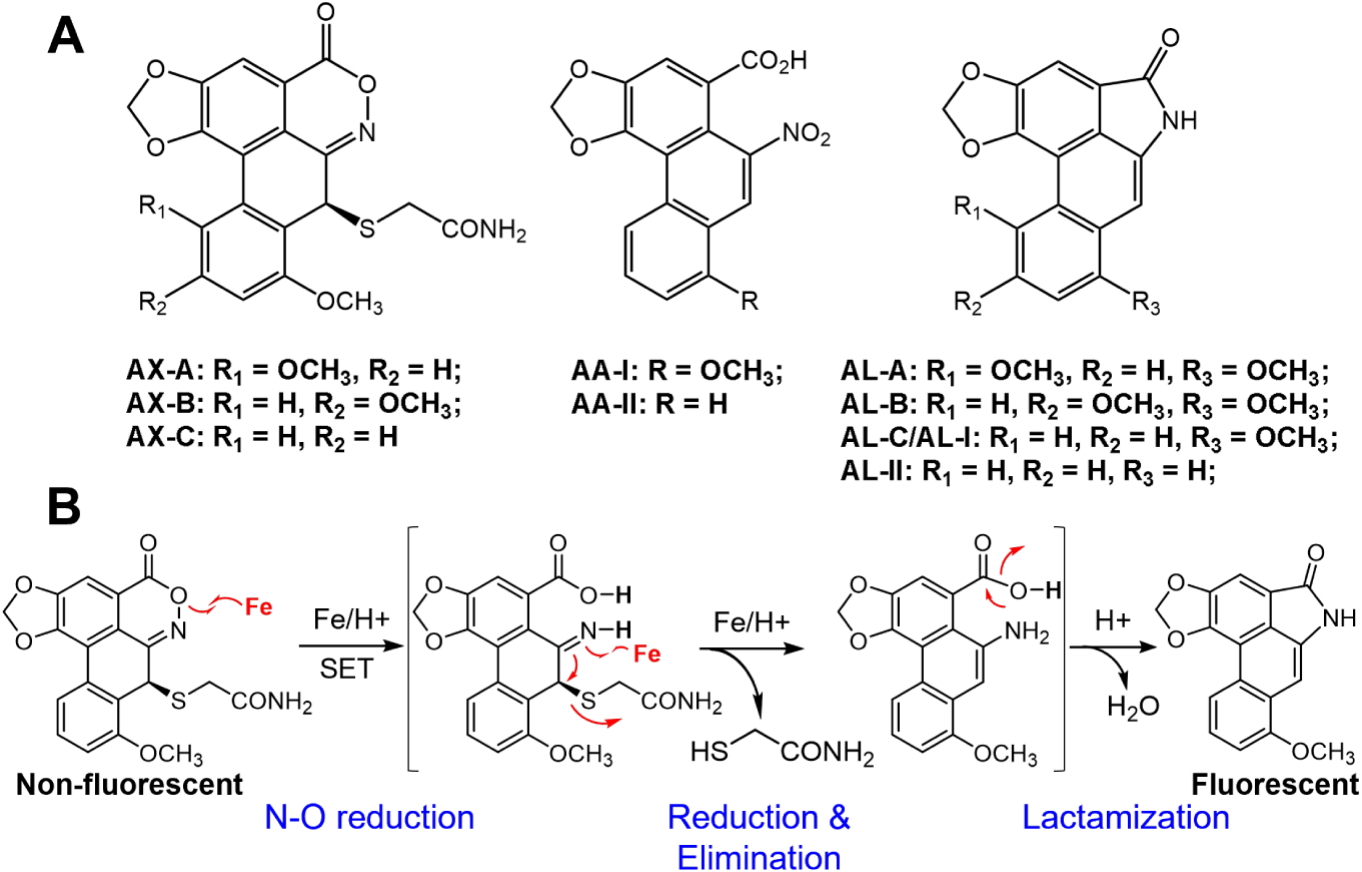
(A) Chemical structures of aristoloxazines, aristolochic acids, and aristolactams. (B) Reaction scheme shows the conversion of non-fluorescing aristoloxazine C (AX-C) by Fe/H^+^-treatment to strongly fluorescing aristolactam I/aristolactam C (AL-I or AL-C).

Despite their strong antimicrobial properties,^1,2^ emerging evidence indicates that AXs possess significant cytotoxicity and neurotoxicity,^3^ posing unintentional exposure risks to consumers. Therefore, understanding the distribution of AXs in various herbal medicines is crucial for informing consumers, assessing health risks, and guiding policy decisions. However, comprehensive data on AXs in herbal products and analytical methods for their detection are currently lacking in the literature.

This study aims to develop a sensitive and selective high-performance liquid chromatography (HPLC)-based analytical method for the determination of AXs. Given their structural similarity to aristolactams (ALs; Figure 1), a highly fluorescent derivative of AAs, our initial focus was on detecting AXs using a fluorescence detector (FLD), which is one of the most sensitive analytical methods. However, AXs are non-fluorescing which precluded their direct detection by HPLC–FLD. To overcome this challenge, we employed an iron powder/acid (Fe/H^+^) reduction condition, previously shown to effectively convert non-fluorescing AAs to strongly fluorescing ALs (Figure S1).^14–16^ Remarkably, this approach yielded a highly fluorescent derivative of AXs that was subsequently characterized as ALs for HPLC–FLD analysis.

Our initial study is to optimize the reduction conditions for the efficient conversion of AXs to ALs. Subsequently, the simultaneous analysis of AXs, ALs, and AAs, in a single HPLC–FLD run, can be achieved by coupling the online post-column reduction using an iron powder-packed microreactor. After validating the proposed method for accuracy and precision, it has been successfully applied to analyze AXs, ALs, and AAs in *Asarum* and *Aristolochia* herbs collected from online and local pharmacies and soil samples from an *Asarum* herb cultivation field. The developed method, in conjunction with mass spectrometric techniques, was also utilized to identify new AXs. Given the evidence that AXs are emerging pollutants in *Asarum* and *Aristolochia* cultivation areas,^4^ we anticipate that the newly developed method will find wide applications in both herbal and environmental sample analyses.

## Materials and Methods

### Chemicals and Reagents

Chemicals and reagents of the highest purity were used. Aristoloxazine A (AX-A), aristoloxazine B (AX-B), aristoloxazine C (AX-C), and aristoloxazine D (AX-D) were extracted from herbal medicines and purified by HPLC, as described previously.^4^ Aristolochic acid I (AA-I), aristolochic acid II (AA-II), aristolactam I (AL-I), and aristolactam II (AL-II) were obtained from Sigma-Aldrich. HPLC-grade methanol and acetonitrile were obtained from Tedia (Fairfield, OH). Reagent water was produced by using a Pall Cascada I laboratory water purification system (Port Washington, NY).

### Instrumental Analyses

HPLC–FLD analysis was conducted on an UltiMate 3000 HPLC system consisting of a binary pump, an autosampler, and an FLD-3400 RS fluorescence detector (Thermo Fisher Scientific; Waltham, MA). UV absorption and fluorescence spectra were acquired on a DAD-3000 diode array detector and an FLD-3400 RS fluorescence detector, respectively. Fluorescence quantum yields were determined on an FS5 spectrofluorometer (Edinburgh Instruments Ltd., Livingston, UK) following manufacturer’s instructions. High-resolution MS, MS/MS, and MS^3^ analyses of AXs and ALs were performed on an Orbitrap Exploris 120 mass spectrometer equipped with a heated electrospray ionization sprayer (Thermo Fisher Scientific; Waltham, MA). LC–MS/MS analyses of AXs were performed on an Acquity UPLC system coupled to a 4000 QTRAP LC–MS/MS system equipped with a standard Turbo V Ion Source (SCIEX; Foster City, CA).

### Fabrication of Inline Microreactors

Microreactors with 4 mm in length and 2.0 mm in internal diameter (i.d.) were fabricated in-house by using SecurityGuard Cartridges (Phenomenex, Torrance, CA). The empty cartridge was packed with 44 μm iron powder and housed in a SecurityGuard Standard cartridge holder as a post-analytical column reactor shown in Figure S2.

### Optimization of Microreactor Parameters

The size of the iron powder packing materials and the amount of acid (delivered in the HPLC mobile phase solvents) were optimized by varying their parameters while analyzing a standard solution mixture of AX-A (250 nM), AX-B (50 nM), AX-C (50 nM), and AX-D (50 nM) by HPLC–FLD.

Given the prior observation that Fe/H^+^-mediated reduction proceeds efficiently at room temperature,^16^ the effects of various sizes (10, 20, 44, and 150 μm) of iron powder packed in microreactors were investigated by monitoring the peak areas of the standard mixture of AXs in HPLC–FLD analysis while keeping other parameters unchanged. Then, the effects of different amounts of trifluoroacetic acid (TFA; 0.02%, 0.05%, 0.1%, 0.15%, and 0.2% *v/v*) in water and acetonitrile in HPLC–FLD analysis were studied while keeping other reactor parameters constant.

### Herbal and Soil Sample Collection and Processing

Herbal samples were purchased online from mainland China or from local pharmacies in Hong Kong. Soil samples were collected from a depth of 0–15 cm from the surface in an *Asarum heterotropoides* cultivation field in mainland China. Soil samples were dried using a freeze-dryer, sieved through a 1 mm sieve, and stored at −20 °C, while herbal samples were stored at room temperature prior to extraction for analysis.

Before analysis, 100 mg of the samples were accurately weighed, mixed with 1.0 mL of extraction solvent (methanol/water/acetic acid: 70:25:5 *v/v*), and sonicated at room temperature for 30 min. The samples were then centrifuged at 16 000 × *g* for 10 min, and the supernatants were transferred into HPLC vials for analysis.

### HPLC–FLD Analysis

Ten microliters of the sample extracts were loaded onto a Waters XSelect CSH C18 column (3.0 × 100 mm, 2.5 μm; Waters Corporation, Milford, MA), which was eluted at a constant flow of 400 μL/min using 0.05% trifluoroacetic acid in water (A) and 0.05% trifluoroacetic acid in acetonitrile (B) as the mobile phase solvents. The solvent was programmed as follows: a linear gradient from 35% to 37% B in 8 min; increasing to 70% B in 10 min; increasing to 100% B in 1 min and holding for 3.5 min; decreasing to 35% B in 1 min; and re-equilibrating at initial conditions for 3.5 min before the next injection.

The HPLC column outlet was connected directly to the microreactor composed of a 4 × 2.0 mm i.d. cartridge packed with 44 μm iron powder enclosed in a SecurityGuard Standard holder before reaching the FLD. The FLD was set with sensitivity (detector gain), excitation, and emission wavelengths at 8, 254 nm, and 490 nm, respectively.

### Calibration and Method Validation

A stock solution mixture of AX-A, AX-B, AX-C, and AX-D (10 μM) was prepared in methanol. From this stock solution, working standards (10, 25, 50, 100, and 300 nM; 5 times higher concentration for AX-A) were prepared by serial dilutions using methanol and analyzed by the developed HPLC–FLD method. Calibration curves were established by plotting the peak areas of the analytes from the HPLC–FLD analysis against their corresponding concentrations in the working standards as depicted in Figure S3. Using a similar method, calibration curves for the AAs and ALs were prepared.

Extraction efficiency of AXs, AAs, and ALs from soil and herbal samples was determined by spiking different amounts of the analyte mixtures onto the samples (Table S1). Quality control (QC) samples of AXs at concentrations of 10, 30, and 90 nM were prepared by serially diluting the same stock solution with blank herbal and soil extracts. The samples were tested seven times within a single day and over 7 days within 2 weeks to assess intraday and interday precision, respectively. The amounts of ALs generated from their corresponding AXs were used to indicate the accuracy of the method.

## Results and Discussion

### Characterization of AXs and Derivatives

AXs are a family of molecules containing a phenanthrene skeleton that have the potential to fluoresce. To prepare for their analyses by HPLC–FLD, the fluorescence properties of AXs were initially assessed. The absorption maxima for AXs—280 nm for AX-A, 284 nm for AX-B, and 282 nm for AX-C—were employed for fluorescence assessment, and no fluorescence was observed by scanning the emission spectra for these compounds. This lack of fluorescence is likely due to the presence of the thioether side chain in AXs, which quenches fluorescence—a phenomenon also observed in many other thiol-containing molecules.^17–20^

Inspired by the detection of strongly fluorescing, non-AA-derived signals with retention times similar to those of AXs when analyzing AAs in *Asarum* and certain *Aristolochia* herbs using our in-house fabricated online reactor on an HPLC–DAD–FLD system in Figure S4, as well as the strong fluorescence of *Asarum* sample extracts treated with Fe/H^+^ in Figure S4D, AX-C was sought to convert into a fluorescing derivative offline using Fe/H^+^. Surprisingly, this reaction yields a strongly fluorescing compound resembling AL-I that emits blue light, as depicted in Figure 2A.

**Figure 2:**
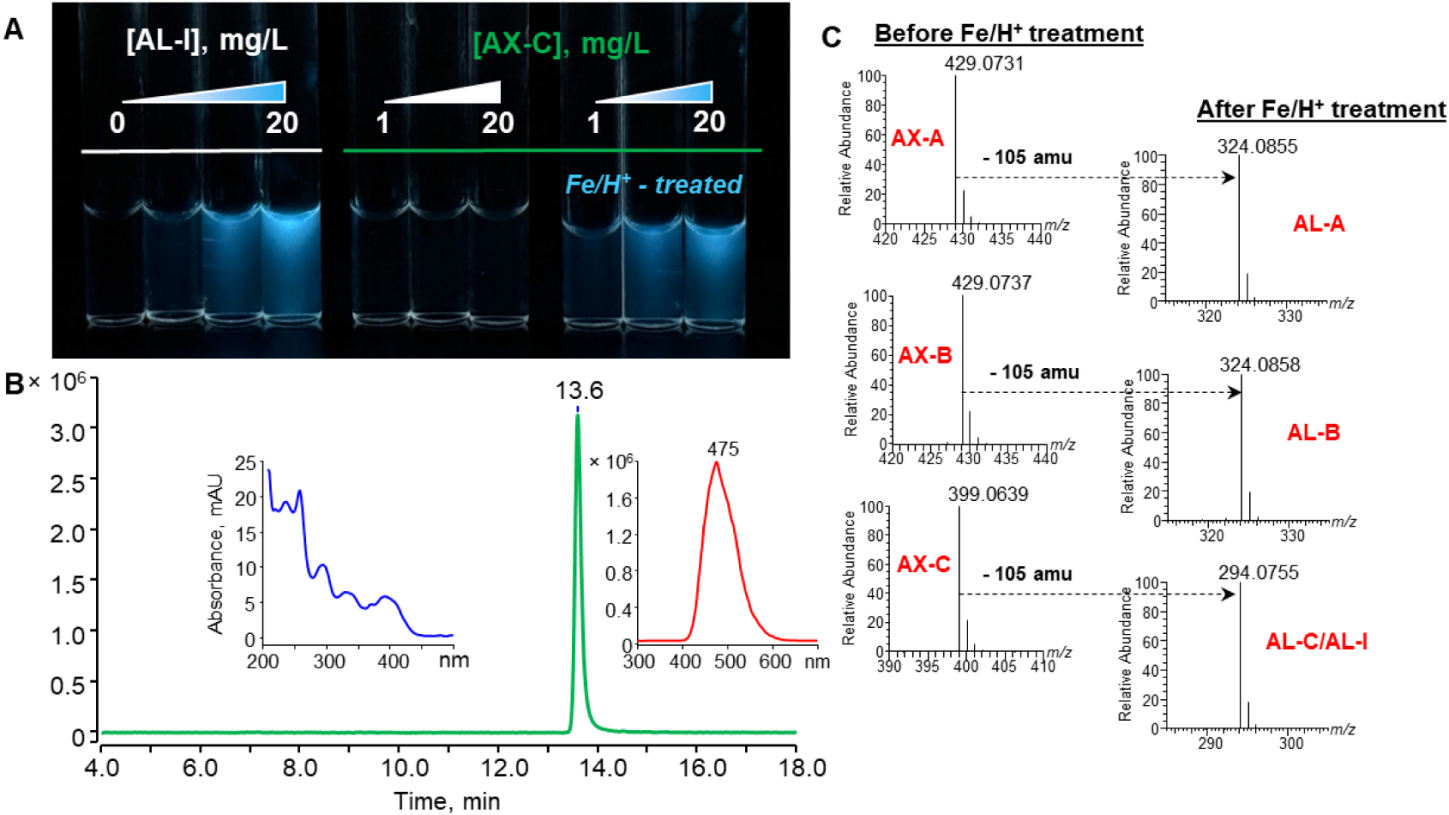
(A) A photograph shows the non-fluorescing AX-C after treatment with Fe/H^+^ emitting a strong blue light with similar luminescence properties as that of AL-I. (B) Chromatogram from HPLC–FLD analysis of AX-C after treatment with Fe/H^+^. The insets show the UV absorption and fluorescence emission spectra of the product from treating AX-C with Fe/H^+^. (C) MS spectra from analyzing AXs before and after treatment with Fe/H^+^.

Figure 2 displays the high-resolution mass spectrometric analysis of the AX-C reduction product, which reveals the rapid production of a major product with [M + H]^+^ at *m*/*z* 294.0755 at high yield. The product matches the theoretical *m*/*z* of AL-I (294.0766) in Table 1, with a chromatographic retention time remarkably similar to that of AL-I (Figure S5). Subsequent confirmatory analyses by tandem mass spectrometry, UV absorption, and fluorescence spectrophotometry confirmed that the product is indeed AL-I as shown in Figure S5. Similar observations of the efficient conversion of AX-A and AX-B to their corresponding ALs were also made upon treatment with Fe/H^+^ in Figure 2. Figure[Fig fig1]B summarizes the general reaction of AXs with Fe/H^+^ to produce ALs.^21,22^

**Table 1:** Mass Spectrometric Data and Fluorescence Quantum Yield of AXs, AAs, and/or ALs

			After treating with Fe/H^+^			
	Measured *m*/*z*		Measured *m*/*z*	Theoretical *m*/*z*	Mass error, ppm	Fluorescence quantum yield, %
**AX-A**	429.0731	**AL-A**	324.0855	324.0872	–5.2	18.1_λ=510_
**AX-B**	429.0737	**AL-B**	324.0858	324.0872	–4.3	28.5_λ=510_
**AX-C**	399.0639	**AL-C/AL-I** ^a^	294.0755	294.0766	–3.7	48.1_λ=470_
**AX-D**	459.0828	**AL-D**	354.0959	354.0978	–5.4	33.6_λ=505_
**AA-I**	359.0877	**AL-I/AL-C** ^a^	294.0773	294.0766	2.4	48.1_λ=470_
**AA-II**	329.0779	**AL-II** ^b^	264.0667	264.0661	2.3	27.2_λ=465_
**AL-I** ^a^	297.0770	N/A	294.0771	294.0766	1.7	48.1_λ=470_
**AL-II** ^b^	264.0666	N/A	264.0660	264.0661	–0.4	27.2_λ=465_

a Represent the same compound AL-C or AL-I.

b Represent the same compound AL-II.

It is important to note that both AA-I and AX-C produced the same product, AL-I, in Figures 2 and S5, upon treatment with Fe/H^+^. If the aforementioned derivatization method was adopted before HPLC–FLD, the resulting AL-I signal might represent a summation of AL-I produced from the reduction of both AA-I and AX-C, in addition to the native AL-I originally present in the sample. This complication is unable to solve by simply analyzing both the pre-column derivatized and underivatized samples as previously reported.^23,24^ To enable the separate identification and quantification of these compounds, Figure 3A displays an online post-chromatographic separation reduction for converting AXs and AAs to ALs. Specifically, AXs and AAs are reduced to fluorescent ALs in an iron powder-packed microreactor for detection in the FLD after they are separated by the analytical HPLC column.

**Figure 3:**
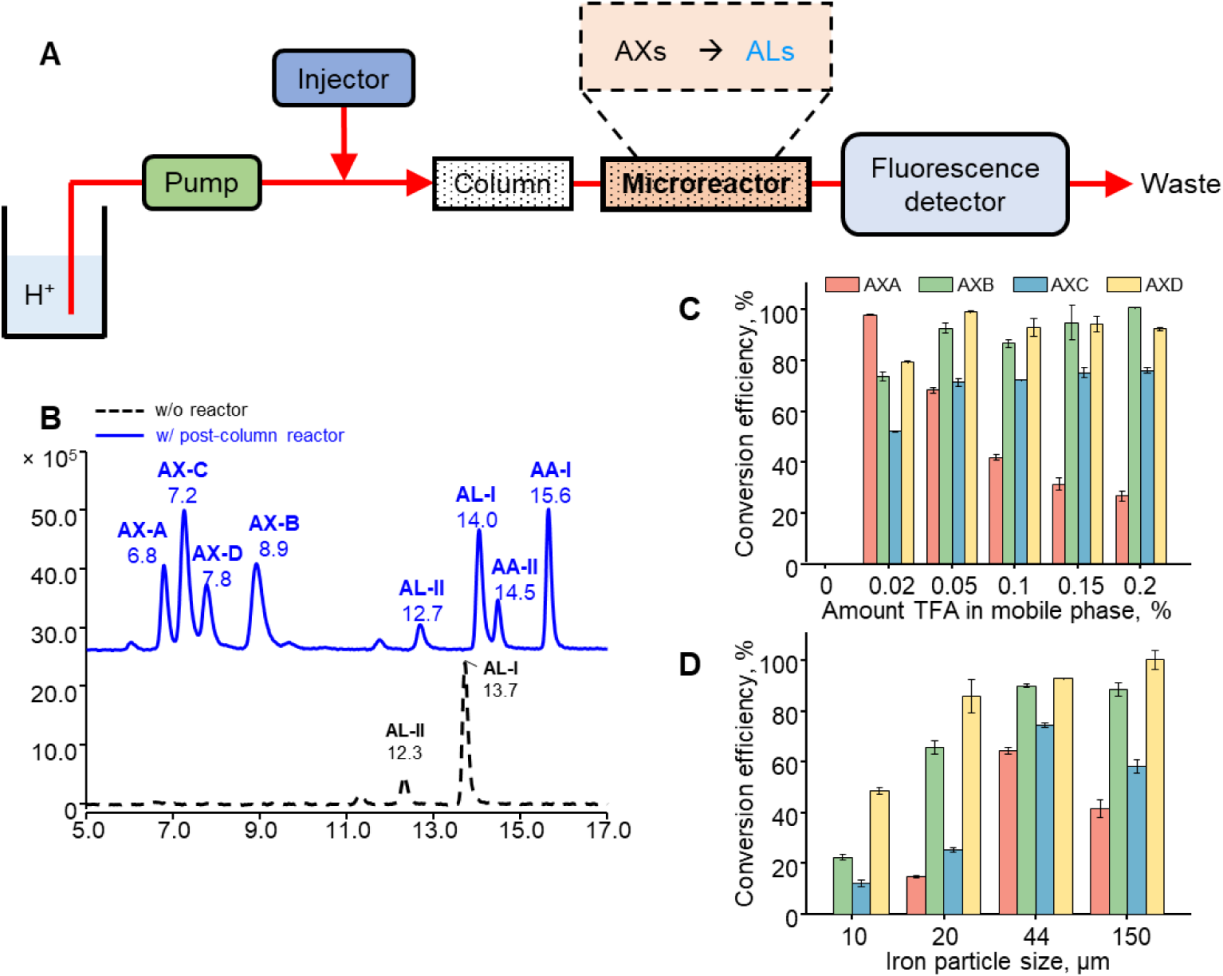
(A) Schematic illustration of using an iron powder-based microreactor for converting non-fluorescing AXs to strongly fluorescing ALs for HPLC–FLD analysis. (B) Typical chromatogram obtained from analyses of a standard solution mixture of AX-A, AX-B, AX-C, AX-D, AA-I, AA-II, AL-I, and AL-II by HPLC–FLD analysis with and without using the microreactor. Effects of changing reactor parameters, such as (C) the amount of TFA in mobile phase solvents and (D) the size of the iron powder packed in the microreactor, on the efficiency of converting AXs to ALs for HPLC–FLD analysis.

### Optimization of Online Reduction Conditions

As previously mentioned, a post-column reduction reactor was used for the derivatization reaction that converts AXs to ALs. An effective post-column reactor should possess the following properties: a small internal volume and high reaction efficiency, minimizing band broadening while achieving a high yield of the product for sensitive detection.

Having demonstrated the high efficiency of converting AXs to ALs using Fe/H^+^, and recognizing the necessity of chromatographically separating AXs, AAs, and ALs prior to FLD detection, the optimal conditions for the post-column reduction of AXs to ALs in an iron powder-packed reactor were investigated, following their chromatographic separation on an analytical column. Increasing the i.d. and length of the reactor could increase the system void volume, which eventually leads to band broadening and spoiling the chromatographic resolution;^16^ as a result, an iron powder-based microreactor prepared by repurposing guard column cartridges with dimensions of 4 mm in length and 2.1 mm i.d. was found to be suitable for this work.

The effects of different concentrations of TFA (0%, 0.02%, 0.05%, 0.1%, 0.15%, and 0.2% *v/v*) in the mobile phase on the conversion efficiency of AXs to ALs in the microreactor after HPLC separation were initially studied. As depicted in Figure 3C, increasing the TFA concentration from 0% to 0.05% *v/v* enhances conversion efficiency for AX-B, AX-C, and AX-D. Further increases in acid concentration to 0.1% or 0.2% *v/v* show little effect on their conversion yields. By contrast, the conversion efficiency of AX-A decreases with the increase in TFA concentration. As such, to compromise the analysis of all four AXs, mobile phase solvents containing 0.05% TFA were selected as the optimal condition in this work.

Additionally, the effects of different iron powder sizes (10, 20, 44, and 150 μm) packed in the microreactor on the conversion efficiency of AXs to ALs were investigated. In Figure 3D, the conversion efficiency of AX-D increases with the increase in the size of the iron powder, while the efficiency of converting AX-A, AX-B, and AX-C increases with the iron powder size and reaches a maximum at 44 μm iron powder with a slight decrement of conversion efficiency observed at 150 μm iron powder. As a result, 44 μm iron powder was chosen for the optimal size of iron powder for packing the microreactor. Under the optimized online reduction conditions, AXs were reduced to ALs with reduction yields above 70% in both medicinal herb and soil matrices as shown in Tables 2 and S2, indicating that the developed post-column reduction method is highly effective for HPLC–FLD analysis.

**Table 2:** Method Accuracy and Precision of the Developed Online Post-column Microreactor Combined with HPLC–FLD for Analysis of AXs in Herbal Sample Extracts

						MDL	
	Amount AX spiked, nM	Amount AL detected, nM^a^	Yield, %^b^	Intraday precision, %^c^	Interday precision, %^d^	ng/mL	ng/g^e^
AX-A	57.1	42.9 ± 1.1	75.0 ± 3.2	2.8	12.6	13.7	137.5
	228.6	149.3 ± 2.0	65.3 ± 1.7	2.5	17.6		
	960.0	700.4 ± 7.0	73.0 ± 1.3	1.6	11.7		
AX-B	11.9	12.9 ± 0.1	108.5 ± 0.8	1.0	6.1	3.0	29.7
	47.6	40.1 ± 1.6	84.2 ± 4.1	3.0	13.4		
	200.0	189.9 ± 3.0	95.0 ± 1.6	1.4	16.7		
AX-C	16.4	12.4 ± 0.3	75.5 ± 2.1	1.4	5.7	1.8	18.1
	49.2	35.2 ± 0.5	71.4 ± 1.8	1.2	13.3		
	196.8	141.3 ± 0.3	71.8 ± 0.2	0.4	14.1		
AX-D	15.1	15.8 ± 0.3	105.1 ± 1.7	3.0	8.7	2.6	26.3
	60.3	67.3 ± 0.4	111.6 ± 0.6	0.5	6.9		
	253.3	260.0 ± 5.2	102.6 ± 2.0	1.5	13.6		

a The data represent mean ± SD of three independent experiments.

b The data represent concentrations quantified by calibration curves established using aristolactam standards.

c The data represent RSD for seven independent experiments conducted on the same day.

d The data represent RSD for seven independent experiments conducted on seven different days over 2 weeks.

e The method detection limit estimated when 100 mg of the sample was extracted using 1.0 mL of extraction solvent.

### Method Validation

The intraday and interday reproducibility of the developed post-column online reduction method for converting AXs to ALs for HPLC–FLD analysis was evaluated by repeatedly analyzing blank extracts of pooled soil samples and pooled medicinal herb samples spiked with a mixture of AX-A, AX-B, AX-C, and AX-D at three concentration levels. The analyses were conducted within a single day and over 7 days across 2 weeks, yielding reproducibility results of less than 18% RSD (Tables 2 and S2). These results indicate that the proposed method is precise and suitable for the intended analysis. Notably, the matrix effect was minimal for both soil and medicinal herb sample analyses (Figure S6), due to the high selectivity of FLD detection.

Furthermore, the performance of the method was deemed acceptable, with the relative standard deviation (RSD) of the QC analyses remaining below 19% after around 165 injections in 4 days using the same microreactor, as shown in Figure 4A and that for different microreactors below 8% (Figure 4B). The detection limits, defined as the amount of AXs in a blank herbal sample extract that generates an analytical signal three times that of the noise level in the HPLC–FLD analysis, were found to be 13.7 ng/mL for AX-A, 3.0 ng/mL for AX-B, and 1.8 ng/mL for AX-C as shown in Table 2. The data were converted to 137.5, 29.7, and 18.1 ng AX-C/g medicinal herb samples. Slightly lower detection limits were observed for the three AXs in a blank soil sample matrix potentially due to the less complex matrix in soil as compared to that of the medicinal herb extracts (Figure S6).

**Figure 4:**
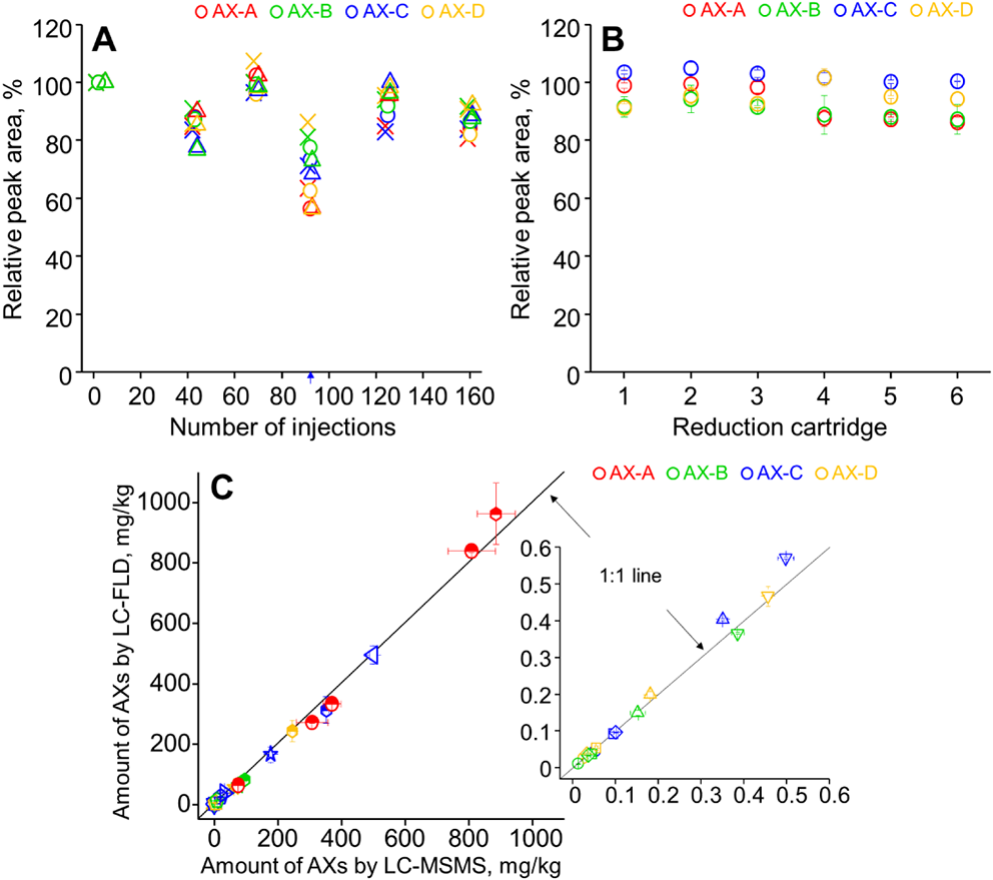
(A) Relative detector response for analyzing standard solution mixtures (at 10 (crosses), 30 (circles), and 90 nM (triangles)) of AX-A, AX-B, AX-C, and AX-D with 165 analyses conducted within 4 days. Cleaning and reactivation of the microreactor were carried out after QC samples indicated with a blue arrow. (B) Relative detector response for analyzing a standard solution mixture (30 nM) of AX-A, AX-B, AX-C, and AX-D using six different microreactors prepared by the same procedure. (C) Comparison of AX concentrations in herbal and soil samples using the developed HPLC–FLD and LC–MS/MS methods (*r* = 0.99). Each symbol represents one sample. The same symbol type in different colors indicates different AXs detected within that sample: AX-A (red), AX-B (green), AX-C (blue), and AX-D (orange). The inset in (C) shows the samples at lower concentration levels. Detailed data are listed in Table S3.

#### Application in Herbal Medicines and Soil Sample Analysis

After the accuracy and precision of the developed method were validated and the extraction efficiencies corrected, the method was applied to analyze medicinal herbs and soil samples. Specifically, a total of 111 herbal medicines from the *Asarum* (62 samples) and *Aristolochia* (49 samples) genera were tested. The results indicate in Table 3, for the first time, a large number of positive samples and high levels of AXs in plants of the *Asarum* genus, particularly with AX-C were detected in 97% of the *Asarum* samples with concentrations reaching as high as 0.5 mg/g in an *Asari Radix et Rhizoma* sample that was purchased from mainland China. Because *Asarum* plants are widely used as herbal medicines for treating a variety of human diseases, including cough and SARS-CoV-2, these results unmasked a previously unaware genotoxin exposure pathway that warrants the attention of the general public and regulatory agencies.

**Table 3:** Concentrations of AX-A, AX-B, AX-C, AX-D, AA-I, AA-II, AL-I, and AL-II in 111 Medicinal Herb Samples

		AX-A	AX-B	AX-C	AX-D	AA-I	AA-II	AL-I	AL-II
*Asari Radix et Rhizoma* ^a^ (*n* = 22)	number of positive samples	0	21	21	20	10	0	20	0
	mean concentration, mg/kg	n.d.^c^	165.6 ± 94.6	266.6 ± 133.2	5.4 ± 11.5	1.8 ± 4.5	n.d.	18.9 ± 18.2	n.d.
	concentration range, mg/kg	N/A^d^	12.9–335.0	9.6–519.2	1.8–54.1	1.2–21.1	N/A	0.8–80.4	N/A
*Asari Radix et Rhizoma* ^b^ (*n* = 30)	number of positive samples	0	30	30	30	0	0	30	0
	mean concentration, mg/kg	n.d.	201.1 ± 73.6	254.5 ± 101.6	2.7 ± 0.5	n.d.	n.d.	9.9 ± 5.4	n.d.
	concentration range, mg/kg	N/A	5.1–397.4	22.1–418.3	1.9–3.7	N/A	N/A	0.6–22.5	N/A
*Asarum forbesii* (*n* = 10)	number of positive samples	10	10	9	9	8	0	9	0
	mean concentration, mg/kg	665.4 ± 435.0	200.5 ± 190.5	279.7 ± 281.9	30.6 ± 79.4	41.5 ± 41.9	n.d.	953.5 ± 951.9	n.d.
	concentration range, mg/kg	27.1–1437.0	15.7–560.9	37.1–870.5	1.6–242.2	4.4–100.1	N/A	29.5–3127.9	N/A
*Fructus Aristolochiae* (*n* = 9)	number of positive samples	0	0	9	0	9	9	9	0
	mean concentration, mg/kg	n.d.	n.d.	434.5 ± 287.4	n.d.	280.2 ± 152	35.1 ± 8.9	97.9 ± 52.3	n.d.
	concentration range, mg/kg	N/A	N/A	150.7–1022.7	N/A	126.4–556.1	23.4–48.1	33.9–200.6	N/A
*Herba Aristolochiae* (*n* = 11)	number of positive samples	0	0	11	0	11	11	11	0
	mean concentration, mg/kg	n.d.	n.d.	66.1 ± 27.2	n.d.	45.6 ± 27.9	17.1 ± 6.9	34.7 ± 8.1	n.d.
	concentration range, mg/kg	N/A	N/A	33.7–126.6	N/A	19.6–118.1	10.4–28.1	21.6–47.6	N/A
*Radix Aristolochiae* (*n* = 9)	number of positive samples	0	0	8	0	8	8	0	0
	mean concentration, mg/kg	n.d.	n.d.	18.3 ± 11.6	n.d.	1806.5 ± 721.0	242.6 ± 112.3	n.d.	n.d.
	concentration range, mg/kg	N/A	N/A	9.8–40.3	N/A	1620.7–2381.9	158.8–366.9	N/A	N/A
*Herba Aristolochiae Mollissimae* (*n* = 10)	number of positive samples	0	0	0	0	10	10	0	0
	mean concentration, mg/kg	n.d.	n.d.	n.d.	n.d.	1342.0 ± 1107.9	29.5 ± 12.5	n.d.	n.d.
	concentration range, mg/kg	N/A	N/A	N/A	N/A	225.9–2601.7	13.4–51.4	N/A	N/A
*Aristolochia cinnabarina* (*n* = 10)	number of positive samples	0	0	0	0	10	10	0	0
	mean concentration, mg/g	n.d.	n.d.	n.d.	n.d.	4.6 ± 2.2	2.1 ± 0.5	n.d.	n.d.
	concentration range, mg/g	N/A	N/A	N/A	N/A	1.9–8.7	1.3–2.8	N/A	N/A

a *Asari Radix et Rhizoma* purchased online from mainland China.

b *Asari Radix et Rhizoma* purchased at local pharmacies in Hong Kong.

c n.d. signifies not detected.

d N/A signifies not applicable.

The method was also applied to quantitate AXs in surface soil samples collected from a cultivation field of *Asarum heterotropoides* (one of the species for producing *Asari Radix et Rhizoma* ^25^). High levels of AXs were detected in most of the samples with 104 out of 110 listed in Table S4. Notably, AX-C was consistently detected as the most abundant AX in soil samples, in terms of positive rate and concentration. Figure 5 shows typical HPLC–FLD chromatograms obtained from analyzing AXs in a soil sample collected from an *Asarum heterotropoides* cultivation field and an *Asarum forbesii* herb with our post-column microreactor.

**Figure 5:**
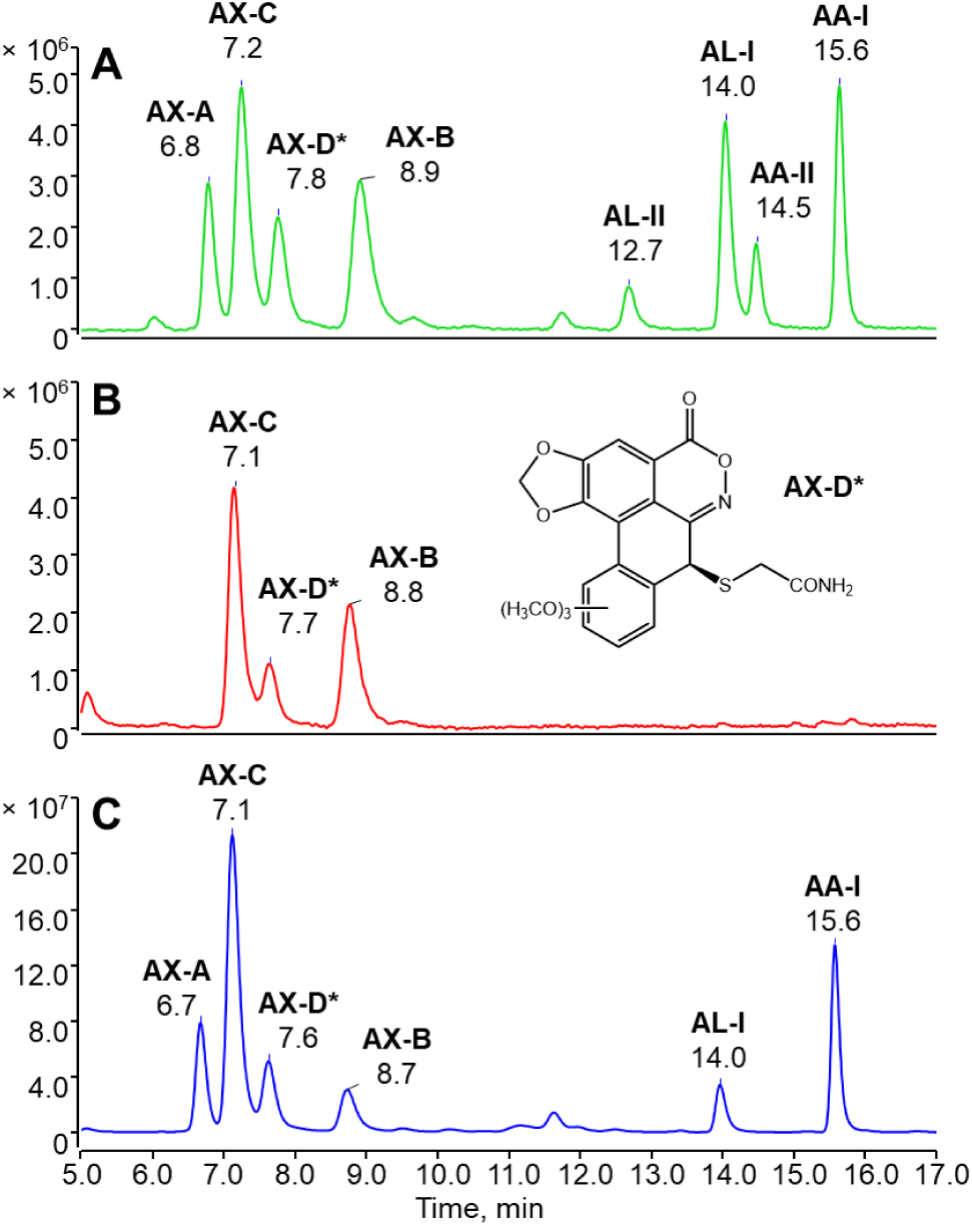
Typical chromatograms obtained from analyzing AXs, AAs, and ALs in (A) a reference standard mixture containing individual analytes at 50 nM (250 nM AX-A), (B) a soil sample collected from an *Asarum heterotropoides* cultivation field, and (C) an *Asarum forbesii* (*Duheng*) extract using the HPLC–FLD analysis combined with the developed online post-column microreactor. AX-A, AX-C, AX-B, AL-II, AL-I, AA-II, and AA-I were eluted at 6.8, 7.2, 8.9, 12.7, 14.0, 14.5, and 15.6 min, respectively. The newly identified AX (AX-D) was eluted at 7.8 min. The proposed chemical structure of the newly identified AX-D is shown in panel B.

It is worth mentioning that high levels of AXs were detected in the collected soil samples. In particular, AX-B, AX-C, and AX-D were found at concentrations exceeding 1100 μg/kg in some samples. As noted in our previous study,^4^ high levels of AXs are released into the soil as unwanted or decaying parts of the herbs break down. Although the transport and accumulation mechanisms of AXs in soil have yet to be elucidated, studies have demonstrated that AXs inhibit the growth of soil microorganisms. Therefore, the widespread presence of this previously unrecognized family of toxicants in soil may pose risks to soil ecology. Future studies should focus on understanding the environmental fate of AXs and developing associated remediation methods.

In a simultaneous analysis, the levels of AAs and ALs in the samples were also quantitated by the same HPLC–FLD analysis. As expected, the analysis revealed generally lower levels of AAs and ALs than AXs in the *Asarum* plants and soil samples from an *Asarum* herb cultivation field as displayed in Tables 3 and S4. In contrast, AAs, particularly AA-I (the well-studied Group I carcinogen classified by the International Agency for Research on Cancer^26–30^ ), were detected at higher levels than AXs in the *Aristolochia* herbs, together with low levels of AL-I. These results demonstrate the applicability of the developed method for the simultaneous analysis of AXs, AAs, and ALs in herbal and soil samples.

#### Comparative Analysis by LC–MS/MS

Following the analysis by the developed HPLC–FLD method, the same herbal and soil sample extracts were analyzed using an LC–MS/MS method, as stated in the Supporting Information. The results from both LC–MS/MS and HPLC–FLD analyses are presented in Figure 4C, with no data differing from each other by 20%, showing excellent agreement in AX levels between the two methods (*p*-values for AX-A: 0.06; AX-B: 0.70; AX-C: 0.52; and AX-D: 0.35). Additionally, both methods yielded similar MDLs for AX-B and AX-C.^4^ This further confirms that the developed method is accurate and suitable for analyzing herbal and soil samples.

#### Screening for New Aristoloxazine Analogs

We have shown that non-fluorescent AXs could be converted to fluorescent ALs after passing through the iron powder-packed microreactor as shown in Figure 3. The produced ALs have molecular weights that are 105 Da smaller than those of their respective precursor AXs. In addition, AXs produce characteristic fragment ions by losing a mercaptoacetamide (C_2_H_5_NOS; 91 Da) moiety in MS/MS analysis as shown in Figure S7; thus, we propose a systematic workflow that is designed to identify new AXs.

Specifically, HPLC–FLD chromatograms obtained from analyses of the same sample with and without a post-column microreactor were compared to identify new potential candidates of AXs. We then scanned for new AXs using a constant-neutral-loss (CNL 91 Da) scan on a triple quadrupole LC–MS/MS system in those positive samples identified in the HPLC–FLD screening. This approach is a powerful tool for identifying unknown compounds within a family that share common fragmentation losses.^31–35^ High-accuracy MS and MS/MS spectra of the new AXs were acquired and studied to identify common fragmentation patterns similar to those of previously identified AXs, e.g., the loss of the mercaptoacetamide moiety (91 Da). Finally, the newly identified AX was reacted with Fe/H^+^ and the reaction products were analyzed by LC–MS and LC–MS/MS to confirm their identities. Figure 6 shows a summary of the strategy used in this study to identify new AXs.

**Figure 6:**
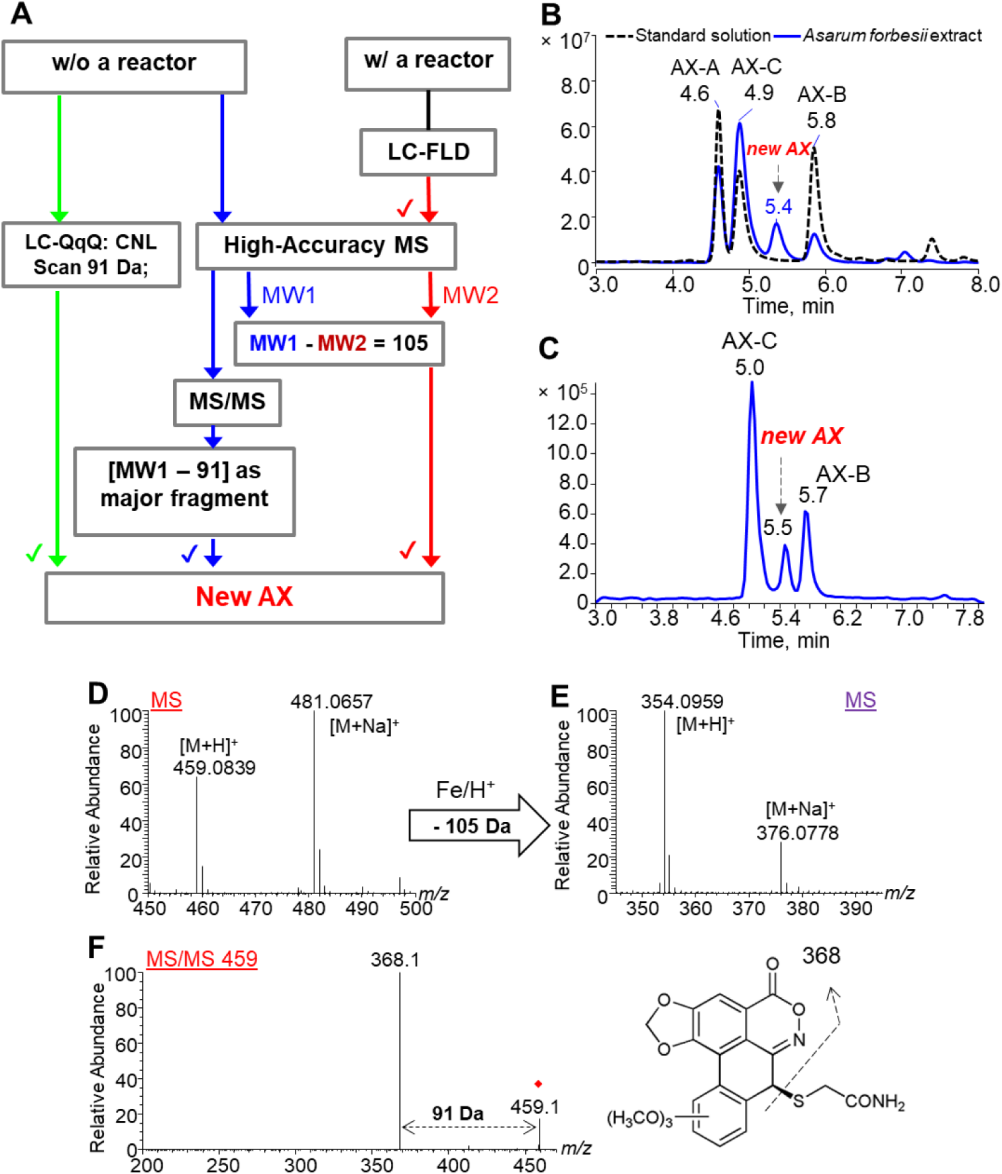
(A) The proposed strategy for identifying new AXs combines HPLC–FLD, LC–MS/MS, and LC–HRMS analyses. Chromatograms from (B) HPLC–FLD analyses of a standard solution containing AX-A, AX-B, and AX-C and a typical *Asarum forbesii* sample extract with a post-column microreactor and (C) constant-neutral loss analysis of 91 Da to search for unknown AXs in a typical *Asari Radix et Rhizoma* (*Xixin*) sample. (D and E) HRMS spectra of the fraction at 5.5 min before and after treatment with Fe/H^+^, and (F) an MS/MS spectrum of the newly identified AX.

The method was used to analyze the 36 herbs of the samples mentioned above for new AXs. In addition to AX-A, AX-B, and AX-C targeted in this study, Figures 6C and S8 depict the CNL analyses of *Xixin* and *Duheng* herbs to reveal a new AX (AX-D) with [M + H]^+^ at *m*/*z* 459.0657, which is 30.01 Da larger than AX-A and AX-B, possibly possessing an additional methoxy (−OCH_3_) moiety. The newly identified AX exhibits a fragmentation pattern similar to that of the other AXs as presented in Figure S9, with a fragment loss of 91 Da, yielding a characteristic ion at *m*/*z* 368.0753 and providing further support for its identification.

Finally, the abundance of this newly identified AX candidate was analyzed with the developed HPLC–FLD method coupled with a post-column microreactor. Similar to other AXs, this reaction produced a strongly fluorescing compound, as shown in Figure 5. Figure 6 displays the LC–MS analysis of the reaction mixture, revealing a product with [M + H]^+^ at *m*/*z* 354.0959, differing from the starting material by 105 Da. This is consistent with the findings for AX-A, AX-B, and AX-C when treated with Fe/H^+^, as illustrated in Figure 2. The MS/MS characterization of the reduction product exhibited a spectrum similar to that of the other AXs. Unfortunately, an insufficient quantity of this newly identified AX was obtained for characterization by NMR. Although definitive confirmation by NMR was not feasible due to the low yield, the similar MS/MS fragmentation pattern of this AX with that of AX-A, AX-B, and AX-C as displayed in Figures 6, S7 and S9, along with the production of a product with a mass 105 Da smaller and similar MS/MS spectra to those produced by other ALs from known AXs (shown in Figure S10), supports the identification of a new AX.

## Conclusion

We fabricated a microreactor from used HPLC guard column cartridges for the online post-column conversion of non-fluorescent AXs and AAs to their fluorescent AL derivatives, facilitating sensitive detection by HPLC–FLD. After cross-validation through comparative analysis with LC–MS/MS, the method was applied to analyze medicinal herbs and soil samples. The analysis detected AXs at concentrations as high as mg/g in some herb samples and at sub-mg/g in some soil samples that warrant the attention of the general public and regulatory agencies. As a previously unrecognized family of genotoxins produced abundantly in certain *Asarum* and *Aristolochia* plants, AXs can contaminate arable soil used for cultivating these plants. However, analytical methods for AXs detection are currently lacking. With careful monitoring of reactor performance, such as through quality control (QC) samples, we believe this newly developed method will have wide applications in high-throughput monitoring by regulatory agencies and in studying the environmental fate and bioavailability of AXs. Furthermore, in combination with mass spectrometric techniques, this method was used to identify new AXs in herbal plants, leading to the successful identification of AX-D as a new member in the AX family. With appropriate modifications, such as adjusting the amount of acid used in the microreactor and incorporating additional sample cleanup steps, we believe the method can be extended to analyze a wider range of structurally similar analogs or transformation products, as well as other samples with different matrices. Future work could also explore more durable, commercially available reactor designs and standardized packing protocols for routine applications.

## Supplementary Material



## Notes

Details on HPLC–DAD–FLD, LC–MS/MS, and LC–HRMS analyses. Extraction efficiencies and method validation results. Concentrations of AXs, AAs, and ALs in soil samples. Concentrations of AXs in soil samples and medicinal herb samples determined by the developed HPLC–FLD and LC–MS/MS methods. Optimized ESI and MRM parameters for LC–MS/MS analysis of AXs, AAs, and ALs. A reaction scheme shows the conversion of aristolochic acids by Fe/H^+^-treatment to aristolactams. A photograph showing the analytical column installed with an iron-powder packed post-column microreactor. Calibration curves of AXs, AAs, and ALs established the developed HPLC–FLD method. Chromatograms obtained from HPLC–DAD–FLD analysis, UV and fluorescence emission spectra, MS/MS and pseudo-MS^3^ spectra of the product of AX-C after Fe/H^+^-treatment. Overlaid chromatograms obtained from analyses of AXs, ALs, and AAs in methanol and pooled sample matrices by the developed HPLC–FLD with a post-column microreactor. Positive and negative ionization mode MS/MS spectra and pseudo-MS^3^ spectra of AX-A, AX-B, AX-C, and AX-D. A chromatogram constant-neutral loss analysis of 91 Da to search for unknown AXs in a typical *Asarum forbesii* (*Duheng)* sample. MS/MS spectra of AX-A, AX-B, and AX-C. MS/MS spectra of the products of AXs after Fe/H^+^-treatment (PDF)
