# Supplementary material for: Simultaneous Determination of Aristoloxazines, Aristolochic Acids, and Aristolactams Using HPLC–Fluorescence Detection with a Post-column Microreactor: Application in Identifying New Aristoloxazines
Source: J Agric Food Chem. 2025 Oct 22;73(44):28483–92. doi: 10.1021/acs.jafc.5c11092 (PMC12593372; doi:10.1021/acs.jafc.5c11092)
Supplement: Supplementary file 1 [file jf5c11092_si_001.pdf]

Simultaneous Determination of Aristoloxazines, Aristolochic Acids, and Aristolactams Using HPLC–Fluorescence Detection with a Post-Column Microreactor: Application in Identifying New Aristoloxazines

Man-Lung Chin [a], Martin M.F. Choi [a], Rongbiao Tong [a], Nikola M. Pavlović[b], Wan Chan[a,*]

[a] Department of Chemistry, The Hong Kong University of Science and Technology, Clear Water Bay, Kowloon 999077, Hong Kong

[b] Medical Faculty, University of Niš, Nis 18000, Serbia; Innovation Center, University of Niš, Univerzitetski trg 2, Niš 18106, Serbia; Kidneya Therapeutics, Belgrade 11070, Serbia.

Total page number: 20, including 5 Tables and 10 Figures

[*] Corresponding authors:

E-mail: chanwan@ust.hk; Phone: +852 2358-7370; Fax: +852 2358-1594.

TABLE OF CONTENTS

EXPERIMENTAL. Instrumental analysis. *(Page S4-S5)*

Table S1. Extraction efficiency and reproducibility for AXs in soil and medicinal herb matrices. *(Page S6)*

Table S2. Method accuracy and precision of the developed online post-column microreactor combined with HPLC–FLD for the analyses of AXs in different sample matrices. *(Page S7)*

Table S3. Concentrations of AXs in soil samples and medicinal herb samples determined by the developed HPLC–FLD and LC–MS/MS methods. *(Page S8)*

Table S4. Concentrations of AXs, AAs, and ALs in soil samples collected from an *Asarum heterotropoides* cultivation field. *(Page S9)*

Table S5. Optimized electrospray ionization and multiple reaction monitoring parameters for the analysis of AXs, AAs, and ALs in medicinal herbs and soil samples. *(Page S10)*

Figure S1. Reaction scheme shows the conversion of non-fluorescing aristolochic acids (AA-I, R = OCH_3; AA-II, R=H) by Fe/H^+-treatment to strongly fluorescing aristolactams ((AL-I, R = OCH_3; AL-II, R=H). *(Page S11)*

Figure S2. A photograph shows an analytical column installed with a microreactor housed in a cartridge holder. *(Page S12)*

Figure S3. Calibration curves of AX-A, AX-B, AX-C, AX-D, AL-I, AL-II, AA-I, and AA-II established using the developed method. Non-fluorescing AXs and AAs were converted by the post-column microreactor to strong fluorophores, aristolactams. *(Page S13)*

Figure S4. (A) HPLC–DAD chromatogram obtained from analyzing a standard solution containing AXs, ALs, and AAs. (B) HPLC–FLD chromatogram obtained from analyzing a standard solution containing AXs, ALs, and AAs with and without a post-column microreactor. (C) A HPLC–FLD chromatogram of a typical *Asari Radix et Rhizoma* sample with a post-column microreactor. *(Page S14)*

Figure S5. Chromatograms obtained from analyses of a standard solution of AL-I and AX-C treated with Fe/H^+ conducted on a HPLC–DAD–FLD system monitoring UV absorption at 254 nm and fluorescence emission wavelength at 490 nm (excitation at 254 nm). Tandem mass spectrometry spectra of the reduction product of AX-C after Fe/H^+-treatment and the proposed MS/MS fragmentation. *(Page S15)*

Figure S6. Overlaid chromatograms obtained from analyses of AXs, ALs, and AAs in pure solvent and extracts of a pooled soil sample and a pooled medicinal herb sample by the developed HPLC–FLD with a post-column microreactor. *(Page S16)*

Figure S7. MS/MS spectra showing the characteristic MS/MS fragments of AX-A, AX-B, and AX-C at low collision energy by losing the mercaptoacetamide moiety. *(Page S17)*

Figure S8. A chromatogram constant-neutral loss analysis of 91 Da to search for unknown AXs in a typical *Asarum forbesii* (*Duheng)* sample. *(Page S18)*

Figure S9. MS/MS spectra of AX-A, AX-B, AX-C, and AX-D at (A to D) negative ion mode and (E to L) positive ion mode. (I to L) Pseudo MS^3 spectra obtained from fragmenting source fragments of AXs (losing a mercaptoacetamide moiety). *(Page S19)*

Figure S10. MS/MS spectra of the products of (A) AX-A, (B) AX-B, (C) AX-C, and (D) AX-D after Fe/H^+-treatment. *(Page S20)*

EXPERIMENTAL

Instrumental analyses

HPLC–DAD–FLD analysis was conducted on an UltiMate 3000 HPLC consisting of a quaternary pump, an autosampler, a DAD-3000 diode array detector (DAD), and a FLD-3400 RS fluorescence detector (FLD) connected in series. The analytical column, post-column microreactor, and mobile phase solvents used were same as those used in HPLC–FLD analysis, unless specified otherwise. The solvent was programmed as follows: a linear gradient from 33% (B) to 34% (B) over 8 min; increasing to 80% (B) in 8 min; increasing to 100% (B) in 1 min and holding for 4 min before equilibrating to initial conditions. The flow rate was set at 450 µL/min. The DAD monitored UV absorption at 254 nm and 280 nm while the FLD acquired fluorescent signal at 490 nm with excitation wavelength set at 254 nm.

LC–MS/MS analysis was conducted using an Agilent Eclipse Plus C18 column (3.0 × 100 mm, 1.8 µm; Agilent Technologies; Santa Clara, CA) which was housed in the column oven thermostatted at 40 °C and eluted at 420 µL/min with 10 mM ammonium acetate in water (A) and acetonitrile (B). The solvent was programmed as follows: a linear gradient from 5% (B) to 70% (B) in 6 min, increasing to 100% (B) in 1 min and holding for 4 min before equilibrating to initial conditions. Ten-µL of sample extract containing 25 µg/L benz[*cd*]indol-2(1*H*)-one as internal standard (IS) was injected for the analysis. Optimized electrospray ionization (ESI) and multiple reaction monitoring (MRM) parameters for simultaneous analysis of AXs, AAs, and ALs were summarized in **TABLE S5**. The same column was used for constant-neutral loss analysis and analyses on the HPLC–DAD–FLD system in screening for new AXs in medicinal herb sample extracts to produce chromatograms shown in **Figure 6**.

LC–HRMS analysis was conducted using a Waters XBridge C18 column (3.0 × 30 mm, 3.5 µm) which was eluted using 0.1% formic acid in water (A) and 0.1% formic acid in acetonitrile (B) at 400 µL/min. A simple linear gradient was used starting from 20% (B) and increasing to 80% in 6 min, followed by increasing to 100% in 1 min and holding for 3 min before equilibrating to initial conditions. The LC flow was directed to the mass spectrometer by a two-position valve. The H-ESI sprayer parameters are as follows: spray voltage: 2000 V and -2000 V for positive and negative ion mode; sheath gas, auxiliary gas, and sweep gas: 35, 10, and 10 arb, respectively; ion transfer tube temperature and vaporizer temperature: 220 °C and 320 °C, respectively. The scan range for MS full scan was from *m/z* 100 to 1000 at 60,000 resolution, whereas for MS/MS and pseudo MS^3 experiments, the scan range was dependent on the *m/z* of the precursor ions.

Table S1. Extraction Efficiency and Reproducibility for AXs in Soil and Medicinal Herbs.

		Concentration spiked, ng/g	Concentration found, ng/g	Recovery, %	Precision Intraday, %	Interday, %
Soil	AX-A	200	194.2 ± 11.8	97.1 ± 6.1	10.0	11.7
		1000	970.6 ± 99.5	97.1 ± 10.3	12.2	14.9
		3000	2827.0 ± 164.2	94.2 ± 5.8	4.2	9.6
	AX-B	200	169.6 ± 3.4	84.8 ± 2.0	5.6	8.9
		1000	780.6 ± 97.7	78.1 ± 12.5	15.3	13.9
		3000	2327.5 ± 99.4	77.6 ± 4.3	10.5	12.4
	AX-C	200	171.1 ± 11.8	85.6 ± 6.9	12.1	12.5
		1000	976.6 ± 19.7	97.7 ± 2.0	17.1	13.3
		3000	2853.6 ± 31.0	95.1 ± 1.1	3.9	8.8
	AX-D	200	180.6 ± 16.2	90.3 ± 9.0	14.2	18.7
		1000	873.1 ± 69.3	87.3 ± 7.9	10.7	15.4
		3000	2568.7 ± 248.1	85.6 ± 9.7	9.8	12.9
		Concentration spiked, µg/g	Concentration found, µg/g	Recovery, %	Precision Intraday, %	Interday, %
Medicinal herb	AX-A	283	261.6 ± 29.5	92.4 ± 11.3	3.5	15.1
		736	590.3 ± 17.5	80.2 ± 3.0	2.0	9.3
		1492	1073.3 ± 7.7	71.9 ± 0.7	7.7	9.0
	AX-B	104	86.4 ± 8.5	83.1 ± 9.8	7.0	4.0
		711	414.2 ± 77.2	58.3 ± 18.6	12.1	24.7
		1704	1038.3 ± 78.5	60.9 ± 7.6	7.6	7.4
	AX-C	267	205.5 ± 9.2	77.0 ± 4.5	1.0	12.7
		777	480.8 ± 7.3	61.9 ± 1.5	1.3	7.2
		1532	889.4 ± 11.0	58.1 ± 1.2	6.3	17.9
	AX-D	40	28.7 ± 3.3	71.8 ± 11.5	5.8	15.9
		120	103.1 ± 19.3	85.9 ± 18.7	13.4	10.5
		300	240.0 ± 22.7	80.0 ± 9.5	8.8	3.4

Table S2. Method Accuracy and Precision of the Developed Online Post-Column Microreactor Combined with HPLC−FLD for the Analyses of AXs in Pure Solution and Pooled Soil Matrix.

		Amount AX spiked, nM	Amount ALs detected, nM[b]	Yield, %[c]	Precision, %[e] Intra-day	Inter-day	MDL ng/L	ng/g[f]
Reagent blank[a]	AX-A	57.1	40.1 ± 0.7	70.1 ± 1.8	1.5	6.0	4.8	/
		228.6	167.0 ± 0.9	73.1 ± 0.5	0.4	10.5		
		960.0	717.9 ± 3.3	74.8 ± 0.5	1.2	2.5		
	AX-B	11.9	9.4 ± 0.5	79.2 ± 5.1	3.1	5.8	0.9	
		47.6	41.6 ± 1.7	87.5 ± 4.0	2.7	8.5		
		200.0	183.6 ± 2.1	91.8 ± 1.1	2.2	7.7		
	AX-C	16.4	11.8 ± 0.1	72.0 ± 1.0	1.8	5.4	1.1	
		49.2	34.3 ± 0.1	69.7 ± 0.3	0.5	3.1		
		196.8	139.5 ± 0.2	70.9 ± 0.1	0.3	7.8		
	AX-D	15.1	13.6 ± 0.1	90.3 ± 0.9	2.3	5.3	3.0	
		60.3	55.5 ± 0.5	92.0 ± 0.8	2.3	10.1		
		253.3	233.4 ± 7.4	92.1 ± 3.2	2.1	6.9		
Soil	AX-A	57.1	38.8 ± 0.7	67.9 ± 1.9	1.8	11.4	3.6	35.6
		228.6	145.2 ± 2.5	63.5 ± 1.7	1.9	13.1		
		960.0	762.2 ± 11.4	79.4 ± 2.2	4.4	16.1		
	AX-B	11.9	10.2 ± 0.4	86.1 ± 4.2	5.1	9.5	0.7	7.2
		47.6	39.9 ± 0.3	83.8 ± 0.9	2.9	13.8		
		200.0	163.9 ± 6.2	81.9 ± 3.8	3.9	13.5		
	AX-C	16.4	12.2 ± 0.1	74.6 ± 0.1	1.1	7.0	0.8	8.2
		49.2	34.7 ± 0.1	70.6 ± 0.3	0.6	8.2		
		196.8	135.7 ± 0.5	69.0 ± 0.4	0.7	9.9		
	AX-D	15.1	13.9 ± 0.3	92.4 ± 2.4	3.3	8.6	2.3	22.8
		60.3	55.3 ± 0.4	91.7 ± 0.7	1.6	10.7		
		253.3	218.0 ± 4.4	86.1 ± 2.0	3.4	10.3		

[a] In methanol/water (80:20; *v/v*).

[b] The data represent mean ± SD of three independent experiments.

[c] The data represent concentrations quantified by calibration curves established using aristolactam standards.

[d] The data represent relative standard deviation for seven independent experiments conducted on the same day.

[e] The data represent relative standard deviation for seven independent experiments conducted on seven different days over two weeks.

[f] Method detection limit estimated when 100 mg of sample was extracted using 1.0 mL extraction solvent

Table S3. Concentrations of AX-A, AX-B, AX-C, and AX-D in Soil Samples Collected from an *Asarum heterotropoides* Cultivation Field and Medicinal Herb Samples Determined by the Developed HPLC–FLD and LC–MS/MS Methods.

Sample		AX-A	AX-B	AX-C	AX-D
Soil_Grid_#1–1	HPLC–FLD, µg/kg	n.d.	11.2 ± 0.7	46.1 ± 1.6	25.7 ± 1.4
	LC–MS/MS, µg/kg	n.d.	12.5 ± 1.1	52.5 ± 6.0	24.4 ± 0.3
Soil_Grid_#2–1	HPLC–FLD, µg/kg	n.d.	149.9 ± 6.9	403.6 ± 2.2	198.9 ± 8.0
	LC–MS/MS, µg/kg	n.d.	152.7 ± 17.7	350.9 ± 9.5	181.2 ± 7.2
Soil_Grid_#3–1	HPLC–FLD, µg/kg	n.d.	365.9 ± 2.2	570.5 ± 3.7	467 ± 26.6
	LC–MS/MS, µg/kg	n.d.	385.3 ± 14.8	498.5 ± 18.9	456.4 ± 8.9
Soil_Grid_#4–1	HPLC–FLD, µg/kg	n.d.	37.5 ± 1.8	92.7 ± 1.0	54.2 ± 6.8
	LC–MS/MS, µg/kg	n.d.	45.7 ± 7.6	95.0 ± 3.7	54.8 ± 3.6
Soil_Grid_#8–1	HPLC–FLD, µg/kg	n.d.	34.0 ± 0.6	96.6 ± 0.8	38.2 ± 1.9
	LC–MS/MS, µg/kg	n.d.	36.5 ± 4.6	100.8 ± 4.5	33.7 ± 6.2
Asari Radix et Rhizoma D[a]	HPLC-FLD, mg/kg	n.d.	61.0 ± 0.7	164.1 ± 25.6	54.1 ± 1.4
	LC–MS/MS, mg/kg	n.d.	75.6 ± 3.5	178.3 ± 5.5	62.6 ± 6.2
Asari Radix et Rhizoma V[b]	HPLC-FLD, mg/kg	n.d.	11.3 ± 1.3	23.3 ± 1.4	3.7 ± 1.0
	LC–MS/MS, mg/kg	n.d.	9.5 ± 1.1	19.7 ± 0.8	3.9 ± 0.4
Herba Aristolochiae B	HPLC-FLD, mg/kg	n.d.	n.d.	36.6 ± 2.5	n.d.
	LC–MS/MS, mg/kg	n.d.	n.d.	33.2 ± 5.6	n.d.
Fructus Aristolochiae H	HPLC-FLD, mg/kg	n.d.	n.d.	493.8 ± 29.3	n.d.
	LC–MS/MS, mg/kg	n.d.	n.d.	499.1 ± 10.7	n.d.
Asarum forbesii (Root) G	HPLC-FLD, mg/kg	960.7 ± 102.1	80.8 ± 3.6	311.1 ± 43.4	242.2 ± 35.5
	LC–MS/MS, mg/kg	885.6 ± 60.4	96.0 ± 5.3	352.2 ± 9.4	246.2 ± 8.0
Asarum forbesii (Root) I	HPLC-FLD, mg/kg	838.2 ± 6.0	62.0 ± 1.4	331.9 ± 1.3	269.6 ± 3.5
	LC–MS/MS, mg/kg	809.1 ± 75.4	75.5 ± 16.3	370.2 ± 28.1	307.8 ± 49.1

[a] *Asari Radix et Rhizoma* purchased online from mainland China.

[b] *Asari Radix et Rhizoma* purchased at local pharmacies in Hong Kong.

Table S4. Concentrations of AX-A, AX-B, AX-C, AX-D, AA-I, AA-II, AL-I, and AL-II in 110 Soil Samples Collected from an *Asarum heterotropoides* Cultivation Field.

	AX-A	AX-B	AX-C	AX-D	AA-I	AA-II	AL-I	AL-II
number of positive samples	0	56	104	85	11	0	3	0
mean concentration, μg/kg	n.d.[a]	152.8 ± 213	315.6 ± 435.1	188.5 ± 210.5	56 ± 20.8	n.d.	11.3 ± 3.1	n.d.
concentration range, μg/kg	N/A[b]	7.2–1161.1	9.3–2560.8	24–1309.5	47.1–117.1	N/A	9.5–14.9	N/A

[a] n.d. signifies not detected.

[b] N/A signifies not applicable.

Table S5. Optimized Electrospray Ionization (ESI) and Multiple Reaction Monitoring (MRM) Parameters for the Analysis of AXs, AAs, and ALs, in Medicinal Herbs and Soil Samples.

	**Ion monitored**	**MRM transitions**	**Time, ms**	**DP, V**	**EP, V**	**CE, V**	**CXP, V**
AX-A/B	$[M+H]^+$	429/338*	50	60	6	20	24
		429/308	50	50	7	60	27
AX-C	$[M+H]^+$	399/308*	50	60	6	30	27
		399/278	50	30	8	50	6
AX-D	$[M+H]^+$	459/368*	50	40	14	50	15
		459/338	50	50	12	40	15
AA-I	$[M+NH_4]^+$	359/298*	50	50	14	20	18
		359/324	50	40	14	20	21
AA-II	$[M+NH_4]^+$	329/268*	50	60	4	20	6
		329/294	50	60	4	25	6
AL-I	$[M+H]^+$	294/279*	50	80	14	40	6
		294/251	50	80	14	50	6
AL-II	$[M+H]^+$	264/206*	50	70	10	40	9
		264/179	50	80	14	50	6

* MRM transitions that were used for quantification. The optimized ESI parameters are as follows: curtain gas: 10 psi; collision gas: medium; ionspray voltage: 4000 V; source temperature: 500 °C; ion source gas 1 (sheath gas): 30 psi; ion source gas 2 (drying gas): 60 psi.

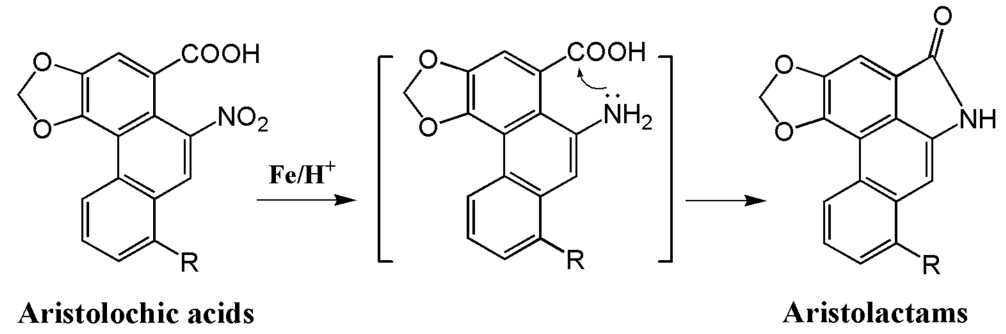

Figure S1. Reaction scheme shows the conversion of non-fluorescing aristolochic acids (AA-I, R = OCH_3; AA-II, R=H) by Fe/H^+-treatment to strongly fluorescing aristolactams ((AL-I, R = OCH_3; AL-II, R=H).

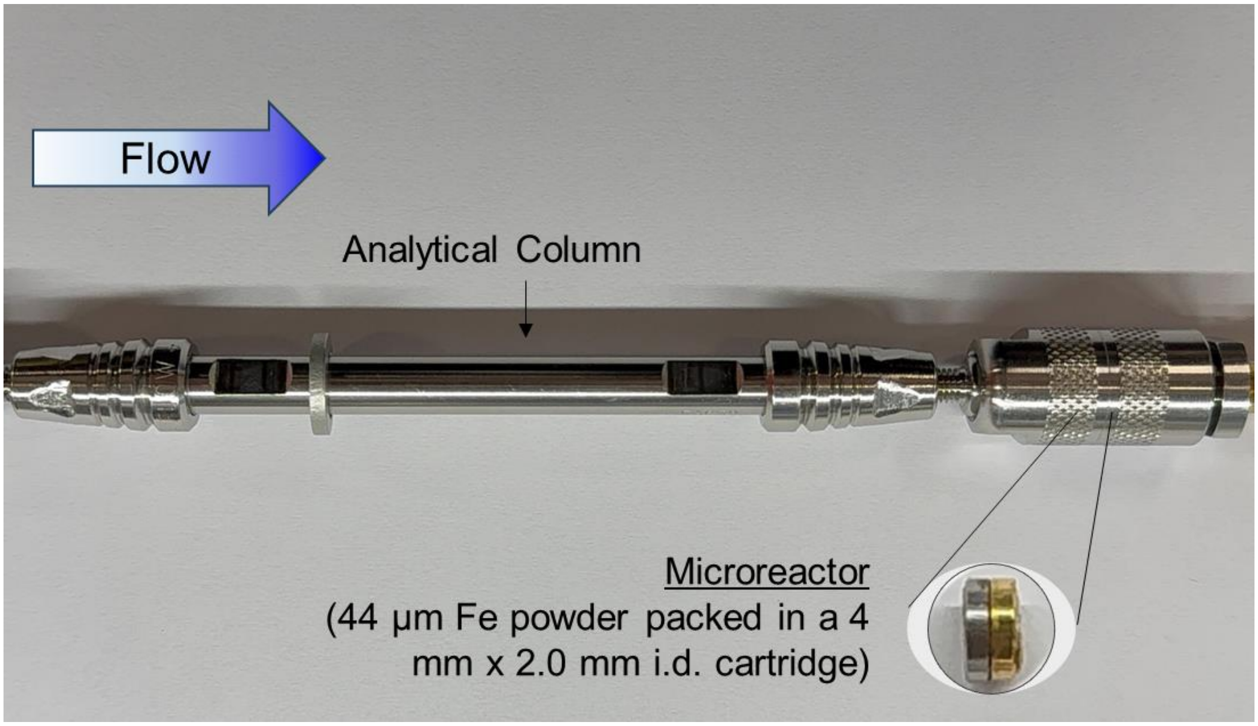

Figure S2. A photograph shows an analytical column installed with a microreactor (a cartridge packed with 44 µm iron powder housed inside the cartridge holder).

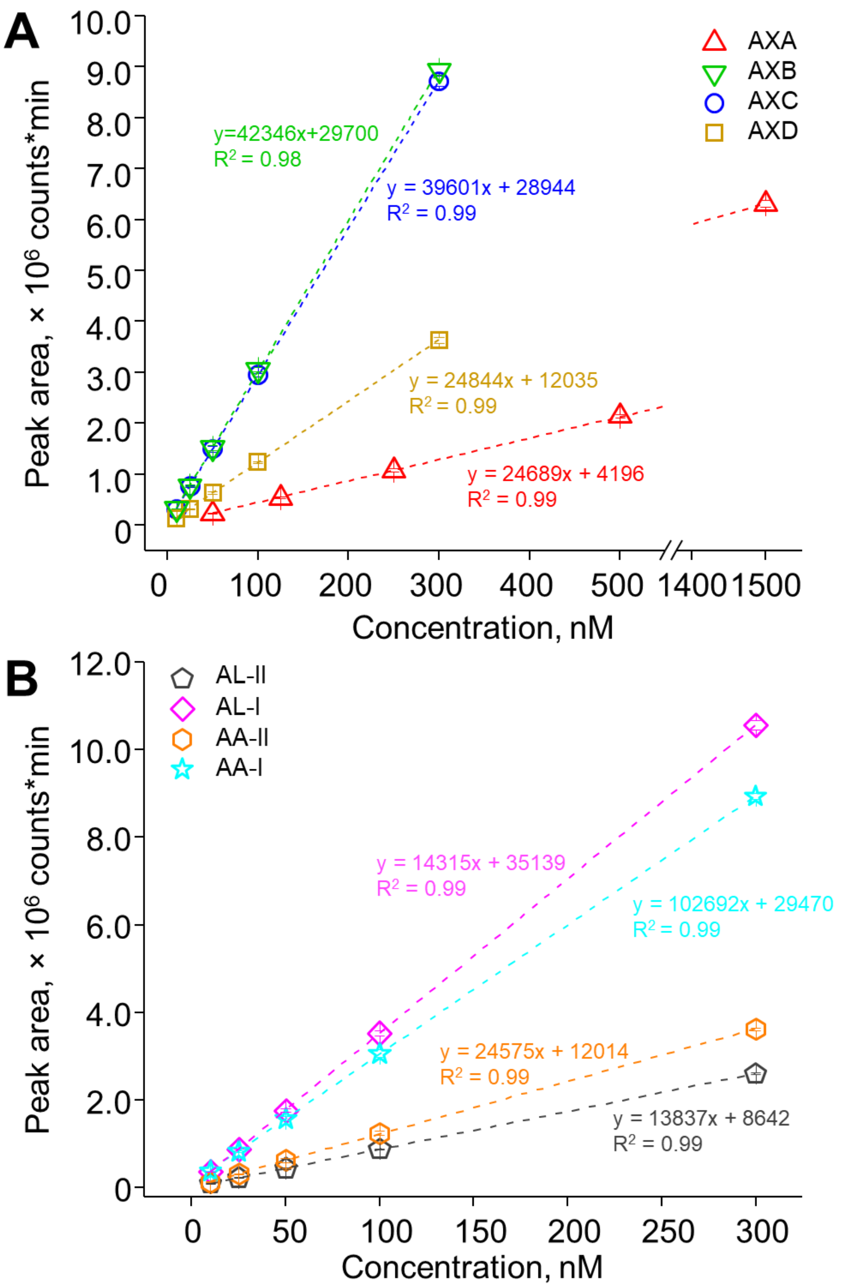

Figure S3. Calibration curves of AX–A, AX–B, AX–C, AX–D, AA–I, AA–II, AL–I, and AL–II established using the developed HPLC–FLD method.

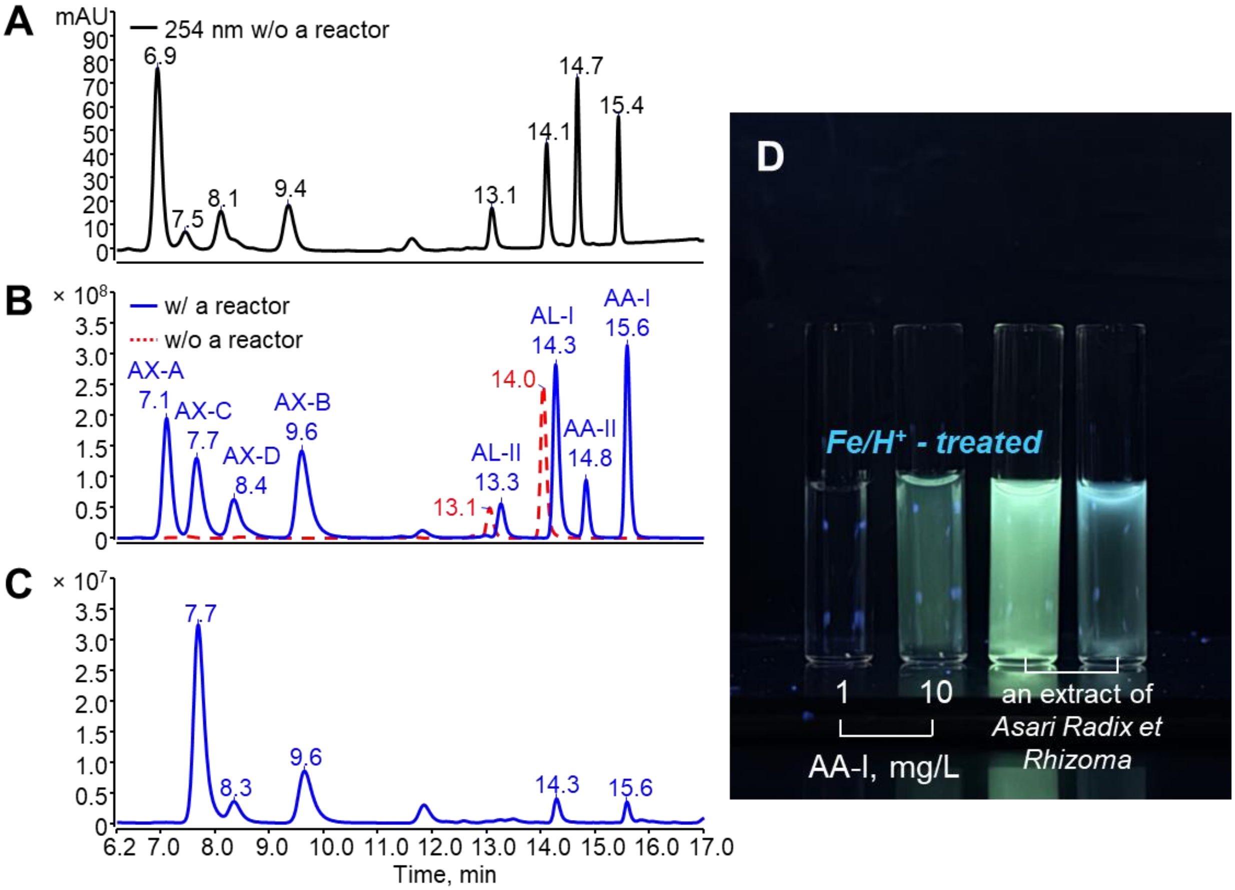

Figure S4. Chromatograms obtained from analyzing (A and B) a standard solution containing a mixture of AX–A, AX–B, AX–C, AX–D, AL–II, AL–I, AA–II, and AA–I with and without a post–column reactor and (C) a typical *Asari Radix et Rhizoma* sample extract with a post–column microreactor on a HPLC–DAD–FLD system. (D) A photograph shows the fluorescence response in Fe/H^+–treated AA–I standard solution and in a typical *Asari Radix et Rhizoma* extract with and without Fe/H^+-treatment.

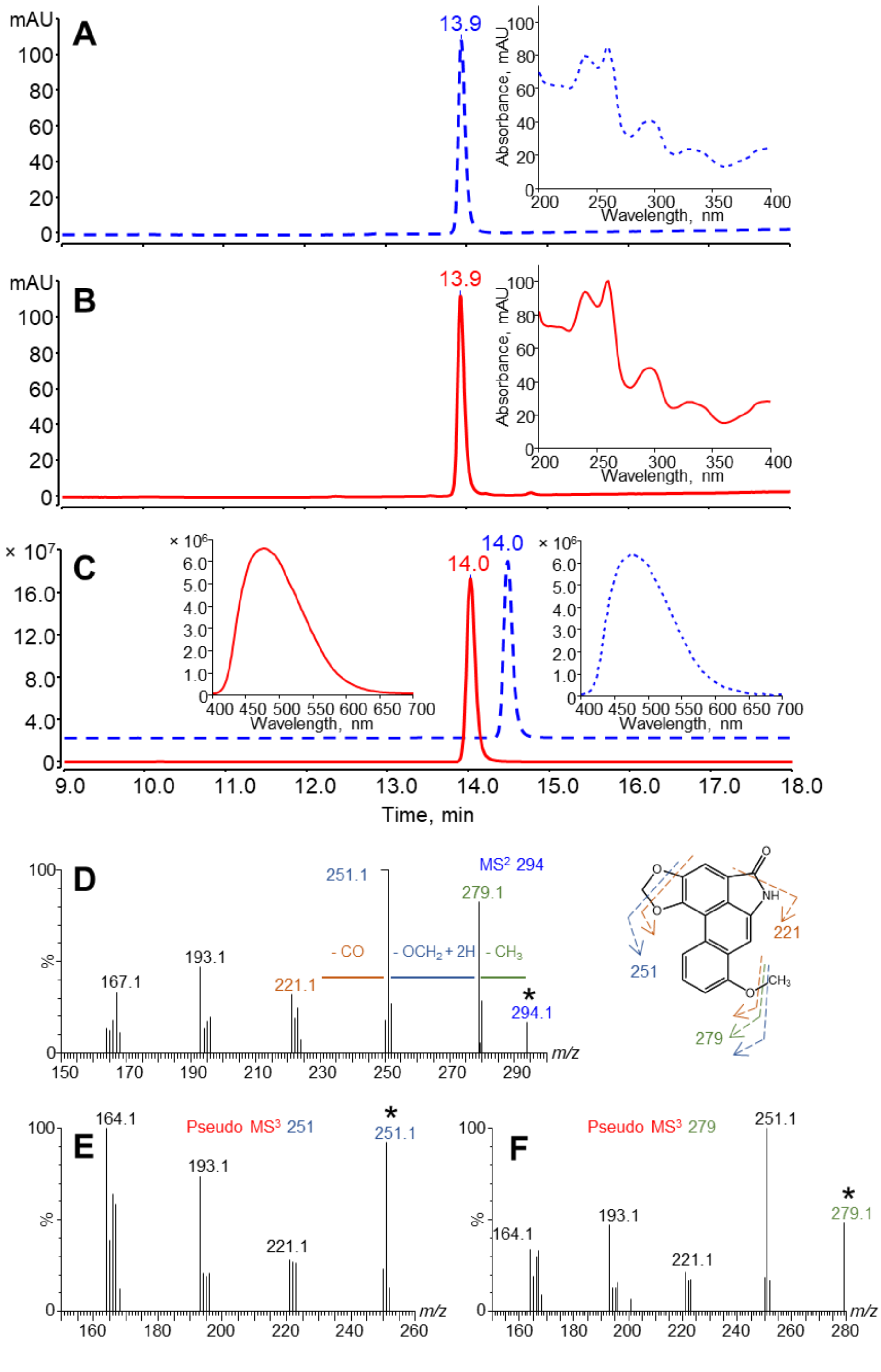

Figure S5. HPLC–UV chromatograms obtained from analyzing (A) a standard solution of AL-I and (B) AX-C treated with Fe/H^+, respectively while (C) is the overlaid FLD channels of the two samples analyzed on a HPLC–DAD–FLD system monitoring UV absorption at 254 nm and fluorescence emission wavelength at 490 nm (excitation at 254 nm). (D) MS/MS spectrum of reduction product of AX-C after Fe/H^+-treatment and the proposed MS/MS fragmentation. (E) and (F) are the pseudo MS^3 spectra of source fragments (*m/z* 251 and *m/z* 279) of the product of AX-C after Fe/H^+-treatment (on a Waters Xevo G2-XS QTof).

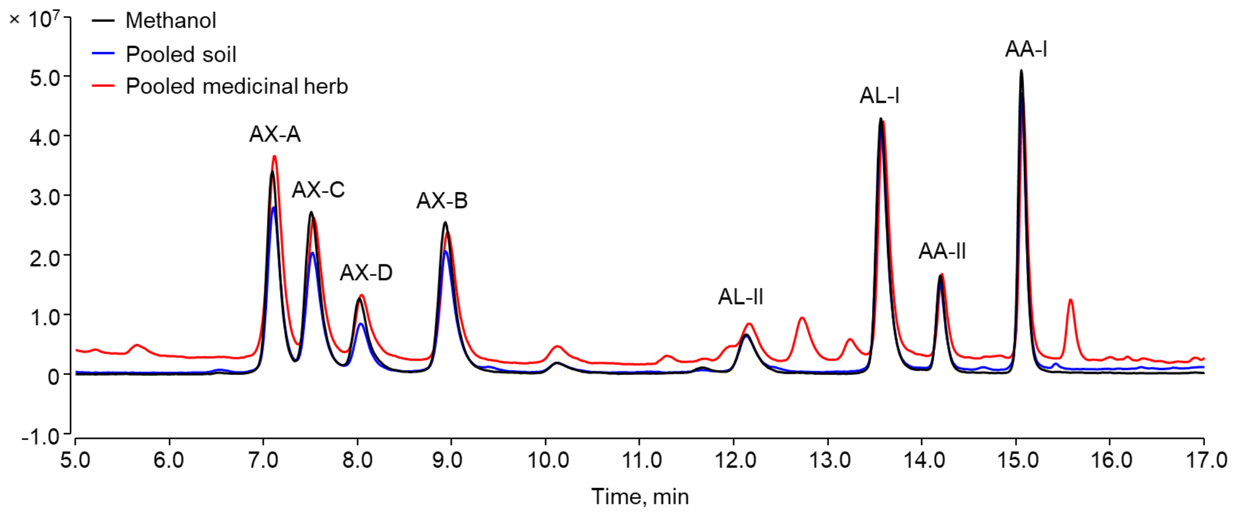

Figure S6. Overlaid chromatograms obtained from analyses of AXs, ALs, and AAs in methanol, an extract of a pooled soil sample, and an extract of a pooled medicinal herb sample by the developed HPLC–FLD with a post-column microreactor.

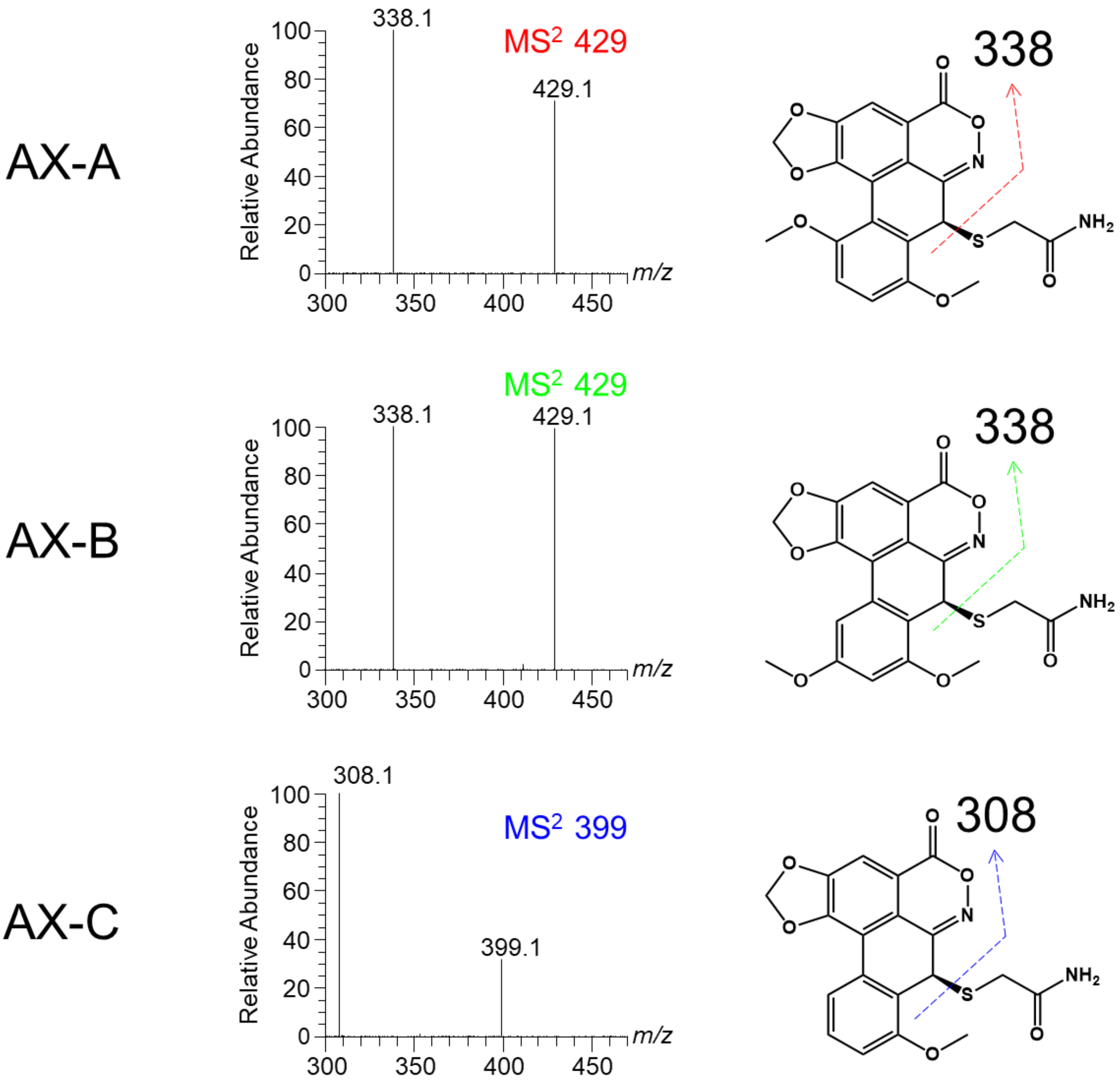

Figure S7. MS/MS spectra showing the characteristic MS/MS fragments of AX-A, AX-B, and AX-C at low collision energy (5 V) by losing the mercaptoacetamide moiety.

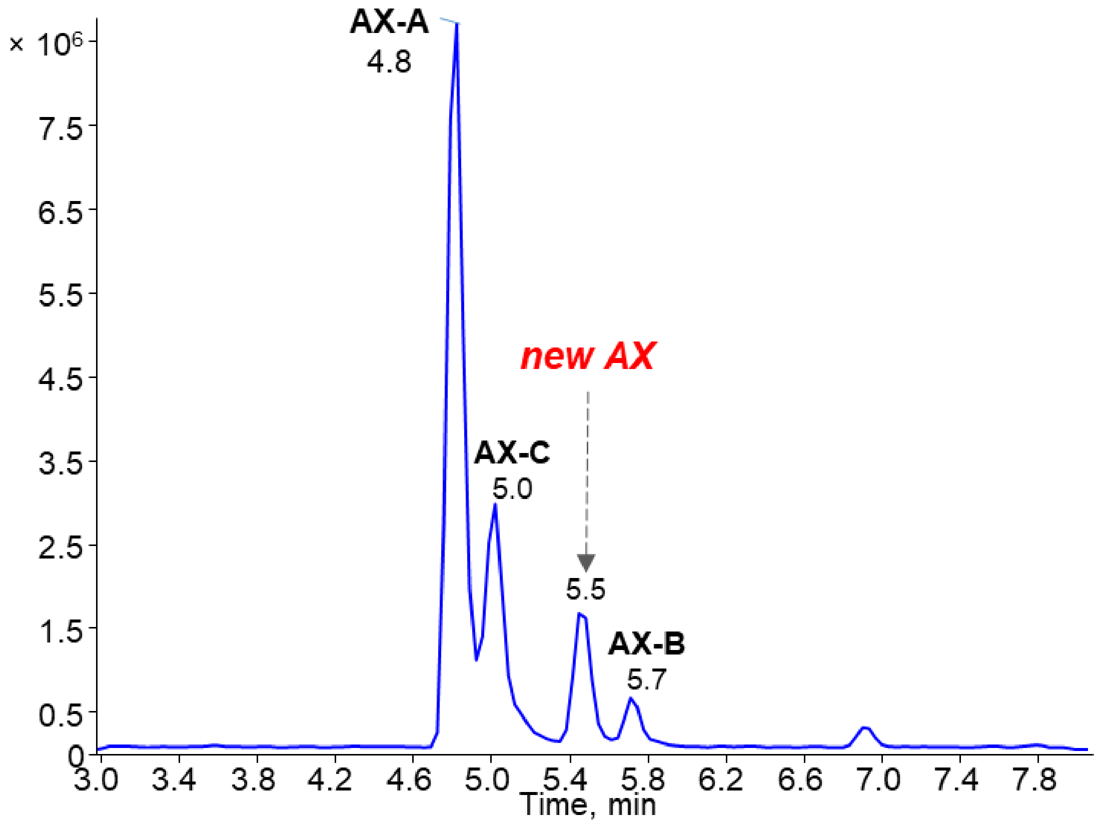

Figure S8. A chromatogram constant-neutral loss analysis of 91 Da to search for unknown AXs in a typical *Asarum forbesii* (*Duheng)* sample.

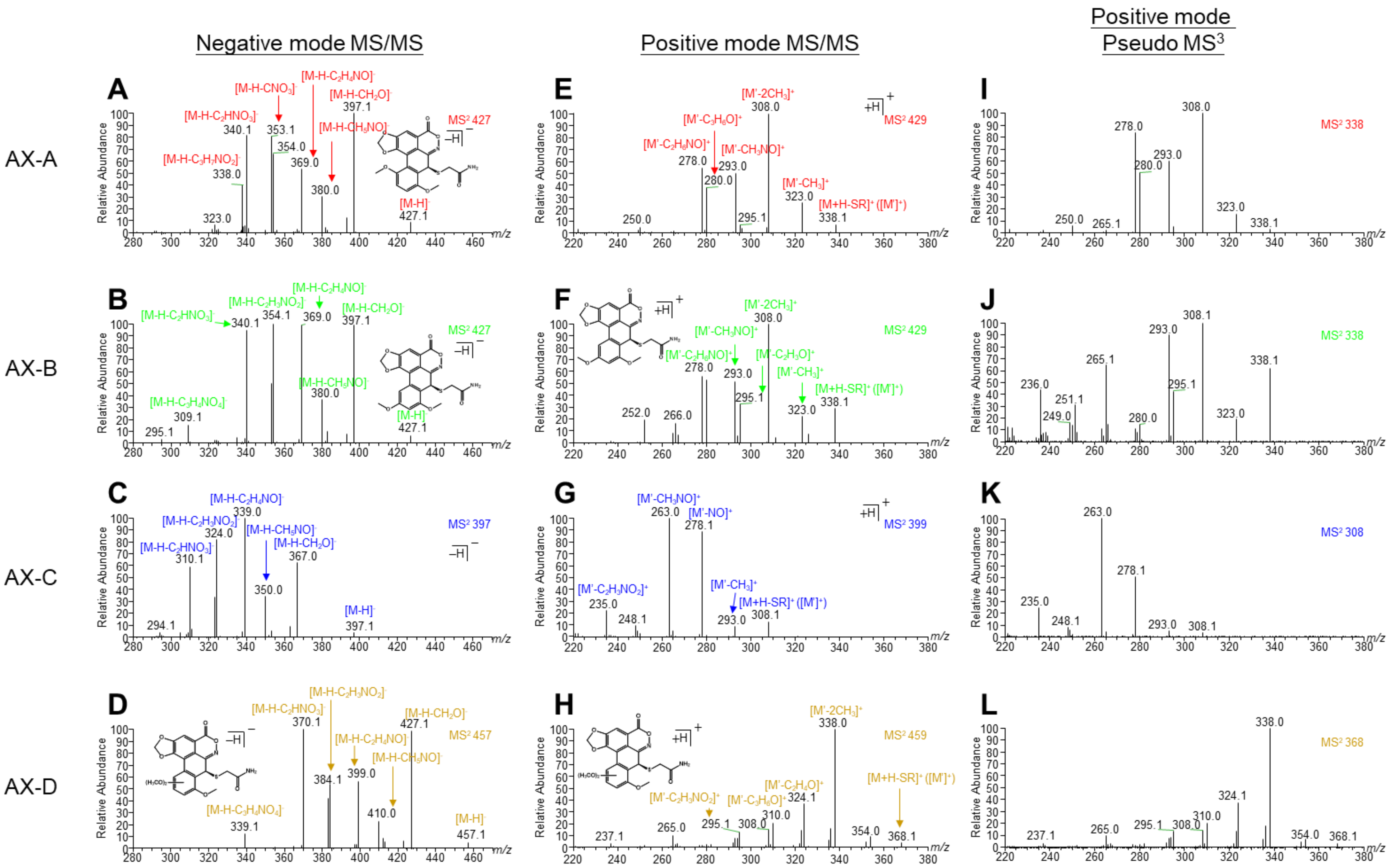

Figure S9. MS/MS spectra of AX-A, AX-B, AX-C, and AX-D at (A to D) negative ion mode and (E to L) positive ion mode. (I to L) Pseudo MS^3 spectra obtained from fragmenting source fragments of AXs (losing a mercaptoacetamide moiety).

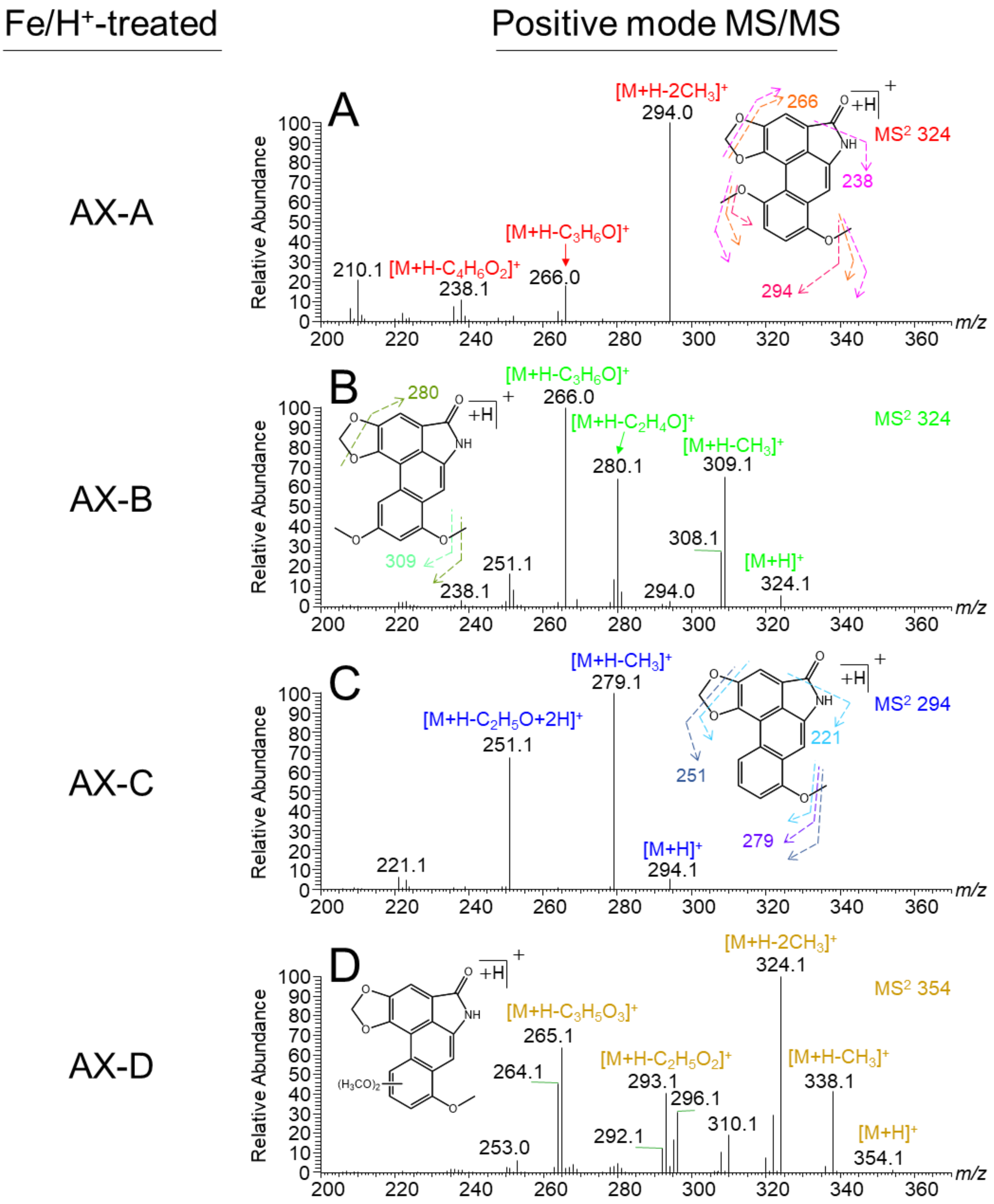

Figure S10. MS/MS spectra of the products of (A) AX-A, (B) AX-B, (C) AX-C, and (D) AX-D after Fe/H^+-treatment.
